# Lymphocyte-Related Immunomodulatory Therapy with Siponimod (BAF-312) Improves Outcomes in Mice with Acute Intracerebral Hemorrhage

**DOI:** 10.14336/AD.2022.1102

**Published:** 2023-06-01

**Authors:** Zhiying Zhang, Yinuo Li, Juyuan Shi, Li Zhu, Yinming Dai, Peiji Fu, Simon Liu, Michael Hong, Jiewen Zhang, Jian Wang, Chao Jiang

**Affiliations:** ^1^Department of Neurology, The Fifth Affiliated Hospital of Zhengzhou University, Zhengzhou, China.; ^2^Department of Neurology, The Second People's Hospital of Zhengzhou City, Zhengzhou, China.; ^3^Medical Genomics Unit, National Human Genome Research Institute, Bethesda, MD 20814, USA.; ^4^Department of Internal Medicine, Sinai Hospital of Baltimore, Baltimore, MD 21215, USA.; ^5^Department of Neurology, People's Hospital of Zhengzhou University, Zhengzhou, China.; ^6^Department of Anatomy, School of Basic Medical Sciences, Zhengzhou University, Zhengzhou, China.

**Keywords:** intracerebral hemorrhage, T lymphocytes, immune-inflammatory response, siponimod, brain injury, neurologic function

## Abstract

Modulators of the sphingosine-1-phosphate receptor (S1PR) have been proposed as a promising strategy for treating stroke. However, the detailed mechanisms and the potential translational value of S1PR modulators for intracerebral hemorrhage (ICH) therapy warrant exploration. Using collagenase VII-S-induced ICH in the left striatum of mice, we investigated the effects of siponimod on cellular and molecular immunoinflammatory responses in the hemorrhagic brain in the presence or absence of anti-CD3 monoclonal antibodies (Abs). We also assessed the severity of short- and long-term brain injury and evaluated the efficacy of siponimod in long-term neurologic function. Siponimod treatment significantly decreased brain lesion volume and brain water content on day 3 and the volume of the residual lesion and brain atrophy on day 28. It also inhibited neuronal degeneration on day 3 and improved long-term neurologic function. These protective effects may be associated with a reduction in the expression of lymphotactin (XCL1) and T-helper 1 (Th1)-type cytokines (interleukin 1β and interferon-γ). It may also be associated with inhibition of neutrophil and lymphocyte infiltration and alleviation of T lymphocyte activation in perihematomal tissues on day 3. However, siponimod did not affect the infiltration of natural killer cells (NK) or the activation of CD3-negative immunocytes in perihematomal tissues. Furthermore, it did not influence the activation or proliferation of microglia or astrocytes around the hematoma on day 3. Siponimod appears to have a profound impact on infiltration and activation of T lymphocytes after ICH. The effects of neutralized anti-CD3 Abs-induced T-lymphocyte tolerance on siponimod immunomodulation further confirmed that siponimod alleviated the cellular and molecular Th1 response in the hemorrhagic brain. This study provides preclinical evidence that encourages future investigation of immunomodulators, including siponimod, which target the lymphocyte-related immunoinflammatory reaction in ICH therapy.

Intracerebral hemorrhage (ICH), the second most common subtype of stroke with high mortality and disability characteristics, can be a devastating and fatal medical emergency [1, 2]. After ICH, the mass effect of the hematoma causes primary brain injury, and the by-products of hematoma degradation promote the progression of secondary brain injury by inducing a series of uncontrollable inflammatory cascades, oxidative stress, etc. [3-7]. Because the clinical benefit of surgical evacuation of hematoma for ICH has not yet been established [8, 9], it is critical to explore alternative strategies to mitigate secondary brain damage by regulating the immune-inflammatory response after acute ICH [3, 5-7].

The immune-inflammatory cascade in the hemorrhagic brain involves the activation of glial cells and the infiltration of circulating immunocytes, including lymphocytes, neutrophils, monocytes/macrophages, etc. [3, 10, 11]. Like innate brain immune cells, circulating immunocytes also play a decisive role in the secondary brain injury and repair process after ICH [12, 13]. Once infiltrated into the injured brain, they produce various cytokines, modulate the neuroinflammatory response, and profoundly influence the severity of brain injury and neurologic outcomes after ICH [6, 12-14]. Although studies suggest that lymphocytes play a bidirectional role in the pathophysiological process of ICH, there is evidence that inhibition of the mobilization of lymphocytes from the peripheral blood to the injured brain may alleviate neuroinflammatory responses and mitigate the severity of brain injury in the acute phase of ICH [15-17]. Therefore, regulation of the mobilization and activation of lymphocytes may represent a promising target for ICH treatment.

T-helper cells (Th) are an essential subset of T lymphocytes that can be recruited to the hemorrhagic brain after ICH [3]. The immune response can be functionally classified as Th1 and Th2 [18]. The Th1 immune response is characterized by an increased release of proinflammatory cytokines including interleukin 1β (IL-1β), interferon-γ (IFN-γ), tumor necrosis factor-α (TNF-α), etc. [19, 20]. The Th2 immune response presents as an increase in the expression of anti-inflammatory cytokines, including IL-4, IL-5, IL-10, and IL-13 [21, 22]. Evidence implies that the Th1 immune response aggravates brain injury, while the Th2 immune response exerts neuroprotective effects after ischemic stroke [21]. Therefore, inhibition of the Th1 immune response or a shift from Th1 to Th2 cytokine production may help treat ischemic stroke. However, few studies have investigated the role of the Th1 immune response in the pathological process of ICH. On activation, normal T cell expressed and secreted (RANTES) and lymphotactin (XCL1) can enhance the immune response under inflammatory conditions [23-25]. Further exploration of the effects of inhibition on the expression of RANTES, XCL1, and Th1-type cytokine production on the severity of brain injury and the functional outcome of ICH is critical.

Immunomodulatory therapies can alleviate early neuroinflammatory responses and improve the prognosis of ICH [5, 6, 26]. As a potential target, sphingosine 1-phosphate (S1P) has shown the ability to regulate the trafficking of immune cells from peripheral blood to the injured brain through the G-protein-coupled S1P receptor (S1PR) after stroke [27, 28]. Evidence has indicated that S1PRs, particularly S1PR_1_, S1PR_2_, S1PR_3_, and S1PR_5_, are widely expressed in lymphocytes, natural killer cells (NK), astrocytes, oligodendrocytes, microglia, and neurons [29-33]. Siponimod (BAF-312) is an S1PR_1/5_ agonist with strong anti-inflammatory effects and has been approved as the second-generation oral drug for the treatment of multiple sclerosis [34, 35]. In addition, studies also suggest that siponimod may be a strong candidate for stroke treatment [16, 36]. However, current studies on the efficacy of siponimod are inconsistent after stroke [16, 36, 37]. Given that there are multiple neuroprotective mechanisms, the effects of siponimod on the neuroinflammatory response, especially the Th1 immune response, warrant further exploration.

In this study, we investigated the potential benefits of siponimod in ICH. We hypothesized that siponimod could alleviate neuroinflammation and improve neurologic function by reducing circulating lymphocyte infiltration and its production after acute ICH. To test this hypothesis, we examined the influence of siponimod on cellular and molecular neuroinflammatory responses, including microglia/macrophage and astrocyte activation, infiltration of T lymphocyte and NK cells, activated T lymphocytes and other immunocytes, Th1-type cytokine production, etc. in the hemorrhagic brain of mice. We further verified the effects of siponimod on T lymphocyte response and Th1-type cytokine production in an anti-CD3 monoclonal antibody (Abs) induced tolerance model after ICH. For early outcomes of ICH, we evaluated the severity of brain injury, brain edema, and swelling in the acute phase of ICH to improve clinical relevance. Furthermore, we also evaluated residual lesion volume, myelin loss, and brain atrophy on day 28 and neurologic deficits on days 1, 3, 7, 14, and 28 after ICH. This is the first study to investigate the impact of S1PR modulators on the infiltration and activation of different subpopulations of lymphocytes and Th1-type cytokine production in the brain.

## MATERIALS AND METHODS

### Animals

One hundred eighty-four young adult male C57BL/6 mice (10-12 weeks, 22-26 g) were obtained from the Laboratory Animal Center of Zhengzhou University (Henan province, China). Mice were housed in individual cages and had free access to food and water in a pathogen-free animal facility of the fifth Affiliated Hospital of Zhengzhou University. The environment was maintained at a suitable temperature (21-25°C), humidity control, and a 12:12 hour light/dark cycle. All experiment protocols were approved by the Animal Care and Use Committee of Zhengzhou University (K2019009). Animal experiments were also carried out according to the ARRIVE guidelines.

### Intracerebral Hemorrhage Mouse Model

The ICH model was established with collagenase injection in the left striatum of mice, as previously illustrated [38, 39]. Mice were briefly anesthetized with 3.0% isoflurane and maintained at 1% isoflurane in 80% nitrogen and 20% oxygen through a nose cone. The mice were then fixed to a stereotactic head frame (RWD Life Science). A 0.6 mm burr hole was drilled with the cranial drill in the left caudate putamen (0.6 mm anterior and 2.0 mm lateral to the bregma). To induce ICH, the 1 μL Hamilton microinjection needle filled with collagenase VII-S (0.075 U in 0.5 μL of saline, Sigma-Aldrich) was slowly lowered 3.2 mm through the burr hole, and collagenase was infused at a rate of 0.1 μL per minute. After completing the infusion, the needle was held for 10 min to prevent backflow and then withdrawn. Sham-operated mice received only an injection of the same dose of saline. The mice were allowed to recover under observation with free access to food and water, and their rectal temperature was maintained at 37.0 ± 0.5°C throughout the operation and recovery periods (until the anesthetic wore off approximately 30-60 min after the operation) [40].

### Treatment regimens and experimental groups

This study includes two-group experimental designs. The first section was designed to investigate the effects of siponimod on immunocyte infiltration, Th-1 immune response, the severity of brain injury, and long-term neurologic function after ICH. The animals in this section were randomly assigned into the following four groups with computer-generated random numbers: the sham group treated with vehicle, the sham group treated with siponimod, the ICH group treated with vehicle, and the ICH group treated with siponimod [41]. Siponimod (BAF-312, MedChemExpress, HY-12335) at a dose of 1 mg/kg dissolved in 5% DMSO with saline was administered intraperitoneally 30 min after surgery and subcutaneously 24 and 48 h after ICH [16, 42]. Vehicle-treated animals were injected with identical volumes of saline in 5% DMSO.

The second section was designed to further elucidate the immunomodulatory effects of siponimod on ICH by inducing tolerance of T lymphocytes with anti-CD3 Abs (clone 145-2C11) [43, 44]. Animals in this part were randomly selected into the four following groups: ICH + IgG isotype control, ICH + anti-CD3 Abs, ICH + IgG isotype control + Siponimod, ICH + anti-CD3 Abs + Siponimod. Siponimod was given similar to the previous section. Anti-CD3 Abs (Invitrogen, 14-0031-86) at a dose of 20 μg diluted in phosphate buffered saline (PBS, 1μg/μL) was administered intraperitoneally with siponimod at 30 min, 24 h, and 48 h after ICH [43, 44]. Isotype controls were injected with 20 μg of hamster IgG diluted in PBS (1μg/μL) (Proteintech, 65210-1-Ig) [43, 44]. The investigator was blinded to the treatment the animals received.

### Tissue processing, the volume of brain lesion, swelling, and atrophy

As bleeding induced by ruptures of the small penetrating arteries in the brain of ICH patients progresses, collagenase-induced bleeding in the rodent brain will last for six hours and relatively stabilize within three days [4, 8, 45, 46]. Additionally, white matter injury in diffusion tensor imaging was prominent in the corpus callosum and internal capsule on day 3 and then partially recovered over time in collagenase-induced ICH mice [46]. In this study, we detected the volume of brain injury (a combination of hematoma and secondary injury area) and brain swelling on day 3 after ICH. Furthermore, there has been evidence that the hematoma resolved completely at 21 days and formed a glial scar 28 days after ICH in mice [41, 47-49]. To enhance clinical relevance in this study, we also measured residual lesion volume, brain atrophy, and white matter damage 28 days after ICH.

After neurologic deficit assessment, mice were anesthetized with isoflurane and experienced transcardial perfusion with PBS followed by 4% paraformaldehyde (PFA) on day 3 or 28 post-ICH. Their brains were removed, kept in 4% PFA overnight, and then transferred to 20% sucrose at 24 h and 30% sucrose at 48 h [50]. After the brain samples sank in sucrose, they were embedded with an optimal cutting temperature compound. The brain was cut into one 50-μm section and twelve 30-μm sections with a cryostat for a total of ten cycles from the level of the olfactory bulbs to the visual cortex. The 50 μm coronal sections spaced 360 μm apart were stained with Luxol fast blue (LFB) for myelin and Cresyl Violet (CV) for neurons to measure brain lesion volume, white matter injury, brain swelling, and atrophy, and the 30-μm sections were stored in cryopreservation solution at -20°C for immuno-fluorescence and LFB staining [51, 52].

Brain lesion volume on day 3 or 28 after ICH was quantified on 50-μm sections stained with LFB/CV using SigmaScan Pro software (version 5.0.0 for Windows; Systat, San Jose, CA, USA; *n* = 10 per group on day 3, *n* = 12 per group on day 28). The areas of the lesion that lack LFB/CV staining indicated the injured range of the brain sections. The volume in cubic millimeters was calculated by the damaged area multiplied by the interslice distance [46, 53].

Brain swelling (*n* = 10 per group) was measured as previously described on day 3 after ICH [41]. Volumes of the ipsilateral and contralateral hemispheres were quantified with SigmaScan Pro software. Brain swelling was calculated as [(ipsilateral hemisphere volume - contralateral hemisphere volume)/contralateral hemisphere volume] × 100% [39, 45]. Although it includes the mass effect of hematoma, it can partially reflect the severity of acute brain damage and the resulting edema after ICH [54].

Brain atrophy (*n* = 12 per group) induced by cell injury and death and dendritic shrinkage in the hemorrhagic hemisphere on day 28 after ICH was calculated as [contralateral hemisphere volume - ipsilateral hemisphere volume) / contralateral hemisphere volume] ×100% [41, 45].

### Brain water content

Animal studies by our group and others have revealed that vascular permeability peaked on day 3 in collagenase-induced ICH [46, 55, 56]. Mice (*n* = 6 per group) were anesthetized with isoflurane and decapitated 72 h after ICH. Intact cerebral tissue was removed and divided into the ipsilateral striatum, the contralateral striatum, and the cerebellum (internal control). The wet weight of each sample (WW) was obtained immediately with an electronic analytical balance. The brain samples were then dried at 100°C in an electric blast drying oven for 24 h and weighed as dry weight (DW). The calculation of the brain water content was as follows: (WW - DW) / WW × 100% [41, 57].

### Evaluation of neurological deficits

Neurological deficits in collagenase-induced ICH animals remain notable at 10 weeks or 2 months post-ICH [57-59]. According to the recommendations of the Initial Stroke Therapy Academic Industry Roundtable (STAIR) and the requirements of the ARRIVE Guidelines for Reporting Animal Research *in vivo* [60, 61], we performed a long-term neurologic deficit assessment in this study. The modified neurologic deficit score (NDS) was graded on a scale of 0-24 (each test was graded from 0 to 4 without deficit as 0 and maximum deficit as 24), and the corner turn test (CTT) was used to test the neurologic deficits of mice on days 1, 3, 7, 14, and 28 after surgery (*n* = 12 per group) [38, 41]. The modified NDS includes body symmetry, gait, circling behavior, front limb symmetry, climbing, and compulsory circling. Mice were excluded from the study if the modified NDS was less than 10 or more than 18 on day 1 after ICH.

For the corner turn test (CTT), mice were placed in a 30-degree angled corner [38, 62]. The mice would have to turn left or right to exit the corner. The choice of turning direction was recorded for each of the ten trials, with at least one minute between the trials. In 10 trials, the percentage of a left turn was used as the CTT score for each mouse.

### Immunofluorescence

The activation of microglia and astrocytes and the infiltration of leukocytes occur very early on in the perihematomal areas following ICH [14, 63, 64]. In the collagenase-induced hemorrhagic brain of mice, microglial activation reached a peak at 3-7 days and the number of reactive astrocytes significantly increased 72 h after ICH [48, 64]. Regarding leukocytes, evidence indicated that the number of neutrophils infiltrated peaked at 3 days in the perihematomal areas after collagenase-induced ICH in animals [65, 66]. Furthermore, CD4^+^ T lymphocytes were the predominant subpopulation of brain infiltrating T lymphocytes at each time point and peaked 4-5 days in the hemorrhagic mouse brain induced by collagenase [14, 15, 67]. This time point partially overlaps with the peak time point of regulatory T cell infiltration (4-7 days after ICH), which plays anti-inflammatory effects in the recovery phase of ICH [14, 15, 67]. Furthermore, microglia and neutrophils also exert neuroprotective effects by promoting brain repair and clearance of the hematoma in the recovery phase of ICH [7, 15, 68]. In this study, we investigated the influence of siponimod on the pro-inflammatory characteristics of infiltrated and innate immunocytes with immuno-fluorescence staining on day 3 after ICH.

Based on our established protocol, three 30-μm sections per mouse with similar areas of lesions were selected and washed in PBS for 50 min for immunofluorescent analysis (*n* = 6 per group) [69]. Sections were blocked in 3% bovine serum albumin for 60 min at room temperature and then incubated with primary antibodies at 4°C overnight. The following primary antibodies were used: rabbit anti-glial fibrillary acid protein (GFAP, Astrocyte marker, 1:200; 16825-1-AP, Proteintech), rabbit anti-ionized calcium-binding adapter molecule 1 (Iba-1, microglial/macrophage maker, 1:1000; 019-19741, Dako), rabbit anti-myeloperoxidase (MPO, neutrophil maker,1:150; ab9535, Abcam), rat anti-CD3 (1:250; sc-18843, Santa Cruz), rat anti-CD4 (1:250; sc-13573, Santa Cruz), mouse-anti CD8-α (1:250; sc-7970, Santa Cruz), mouse anti-CD69 (1:250; sc-373799, Santa Cruz), and rabbit anti-NKp46 (1:1000; PA5-102860, Invitrogen). Subsequently, the sections were washed three times for five min each with PBS and then incubated with secondary antibodies for one hour at room temperature. The following fluorochrome conjugated secondary antibodies were used: goat anti-rabbit 488 (1:1000; A-11034, Invitrogen), goat anti-rabbit 594 (1:10; R37117, Invitrogen), donkey anti-rat 488 (1:1000; A-21208, Invitrogen), and mouse-IgG_k_ anti-mouse 594 (1:100; sc516178, Santa Cruz). After being washed with PBS for 15 min, sections were incubated with 4′,6-diamidino-2-phenylindole (DAPI, 1:100; C0060, Solarbio) for 10 min and washed again with PBS for 15 min at room temperature. Negative controls consisted of identically processed brain sections, except for the incubation step of primary antibodies. Twelve locations (4 fields × 3 sections) in the perihematomal brain region of each mouse were acquired with a ×20 objective on a fluorescence microscope (Ni-U, Nikon). The numbers of positive cells were quantified by Image J (ImageJ 1.4, NIH, USA) and averaged. The results were expressed as positive cells per square millimeter.

### Fluoro-Jade B staining

In the perihematomal region of rodents, the number of ferroptotic and necrotic cells peaks at 72 h after ICH [50, 70, 71]. Fluoro-Jade B (FJB) staining was used to quantify degenerating neurons, as previously illustrated 72 h after ICH (*n* = 6 per group) [72, 73]. We also selected three sections from each mouse to quantify FJB-positive cells, similar to the steps used in immunofluorescence. The stained sections were observed and photographed (4 fields × 3 sections per mouse) under a fluorescence microscope (Ni-U, Nikon) with an excitation wavelength of 450-490 nm. The method used to quantify FJB-positive cells was the same as the measurement for immunoreactive cells. The results were expressed as FJB-positive cells per square millimeter.

### Flow cytometric analysis

Flow cytometric analysis was used to further support the findings on the influence of siponimod on the infiltration and functional changes of T lymphocytes in the hemorrhagic brain detected with immunofluorescence on day 3 after ICH. Single cell suspensions were prepared using an adult mouse brain tissue dissociation kit (130-107-677, Miltenyi Biotec) as previously described [13, 74]. Briefly, mice (*n* = 6 per group) were perfused with ice-cold PBS to remove blood cells from the bloodstream. Each hemorrhagic hemisphere was transferred to a tissue processing tube (RWD Life Science) filled with the mixture of enzyme A and enzyme B from the enzyme digestion kit. Mechanical enzymatic tissue dissociation was performed using the model of M_ABrain_Heater_1 of the Single Cell Suspension Preparation Instrument (DSC-400, RWD Life Science). After dissociation, the homogenate of each brain sample was filtered with a 70 μm cell strainer to remove debris and red blood cells according to the kit protocol. After centrifugation, cells were washed and resuspended in Dulbecco’s phosphate buffered saline (DPBS, 1X). The cell suspension was then stained with PerCP/Cyanine5.5 anti-mouse CD45 (103132, Biolegend), APC anti-mouse CD3 (100236, Biolegend), Brilliant Violet 605™ anti-mouse CD4 (100451, Biolegend), Pacific Blue™ anti-mouse CD8α (100725, Biolegend), APC/Cyanine7 anti-mouse CD19 (115530, Biolegend), FITC anti-mouse NKp46 (137606, Biolegend), and PE anti-mouse CD69 (104507, Biolegend) antibodies at 4°C for 20 min in the dark. Dead cells were excluded using a Zombie Aqua fixable viability kit (423101, Biolegend). Isotype control antibodies coupled with appropriate fluorescein were also used. Immunocytes were gated according to previous reports [12, 67, 75]. Based on the CD45^+^ cell subpopulation, CD3^+^CD4^+^, CD3^+^CD8^+^, CD3^-^NKp46^+^, and CD3^-^CD19^+^ were used to gate Th cells, cytotoxic T lymphocytes (CTL), NK cells, and B lymphocytes, respectively [3, 71]. The geometric median fluorescence intensity (MFI) of CD69 was used in the CD3^+^CD4^+^ cell subpopulation to evaluate Th cell activation [49]. Data on the percentage and MFI of positive cells were acquired from the FACSVerse analyzer (CytoFLEX, Beckman, USA). All results were analyzed with FlowJo (version 10, Treestar, Ashland, Oregon, USA).

### Western blotting

Pro-inflammatory cytokines increase significantly around the hematoma in the acute phase of ICH [6, 7]. For the detection of the molecular inflammatory response, mice (*n* = 6 per group) were anesthetized with isoflurane and decapitated 36 h after ICH [56]. As illustrated previously, the hemorrhagic hemispheres were immediately collected and placed in liquid nitrogen [41]. Total protein was obtained from hemorrhagic hemispheres by lysing tissue with RIPA lysis buffer and PMSF protease inhibitor (RIPA/PMSF, 100:1, Solarbo) and then quantified using the enhanced BCA protein assay kit (P0010S; Beyotime). The protein samples were heated to 99°C for 10 min. Equal amounts of protein were separated by 15% sodium dodecyl sulfate (SDS)-polyacrylamide gel electrophoresis (PAGE) and transferred to polyvinylidene difluoride (PVDF) membranes. The membranes were blocked with 5% nonfat milk for 2 h and then probed with primary antibodies against mouse antilymphotactin (XCL1, 1:1000; sc-514972, Santa Cruz), mouse anti-β-actin (β-actin, 1:6000; 66009-1-Ig, Proteintech), rabbit anti-high mobility group box 1 (HMGB1, 1:800; ab18256, Abcam), rabbit anti-IL-1β (IL-1β, 1:600; ab200478, Abcam), rabbit anti-RANTES (1:600; ab189841, Abcam), and rabbit anti-Interferon-γ (IFN-γ, 1:6000; ab133566, Abcam) at 4°C overnight. The membranes were then washed three times for 10 min each and incubated with appropriate secondary antibodies, goat anti-mouse (1:6000; SA00001-1, Proteintech) or goat anti-rabbit (1:6000; SA00001-2, Proteintech) for 1 h at 37°C. The protein signal was visualized with the ECL chemiluminescence reagent kit (KF001; Affinity) and semiquantitatively analyzed by Image J. Results were expressed as relative density subtracting background values, normalized to a loading control β-actin.

### White matter damage and myelin loss

White matter damage was detected as previously described [41, 46]. We quantified white matter damage (*n* = 12 per group) in 30-um sections stained with LFB on day 28 after ICH. To examine intact myelin in the external and internal capsules, three different stained sections and four fields per section from each mouse were selected and photographed at the same exposure level under light microscopy. The areas covered by the LFB stain from 12 locations per mouse (4 fields × 3 sections) were quantified with Image J software, averaged, and expressed as a percentage of the total area of the white matter examined.

**Figure 1.**
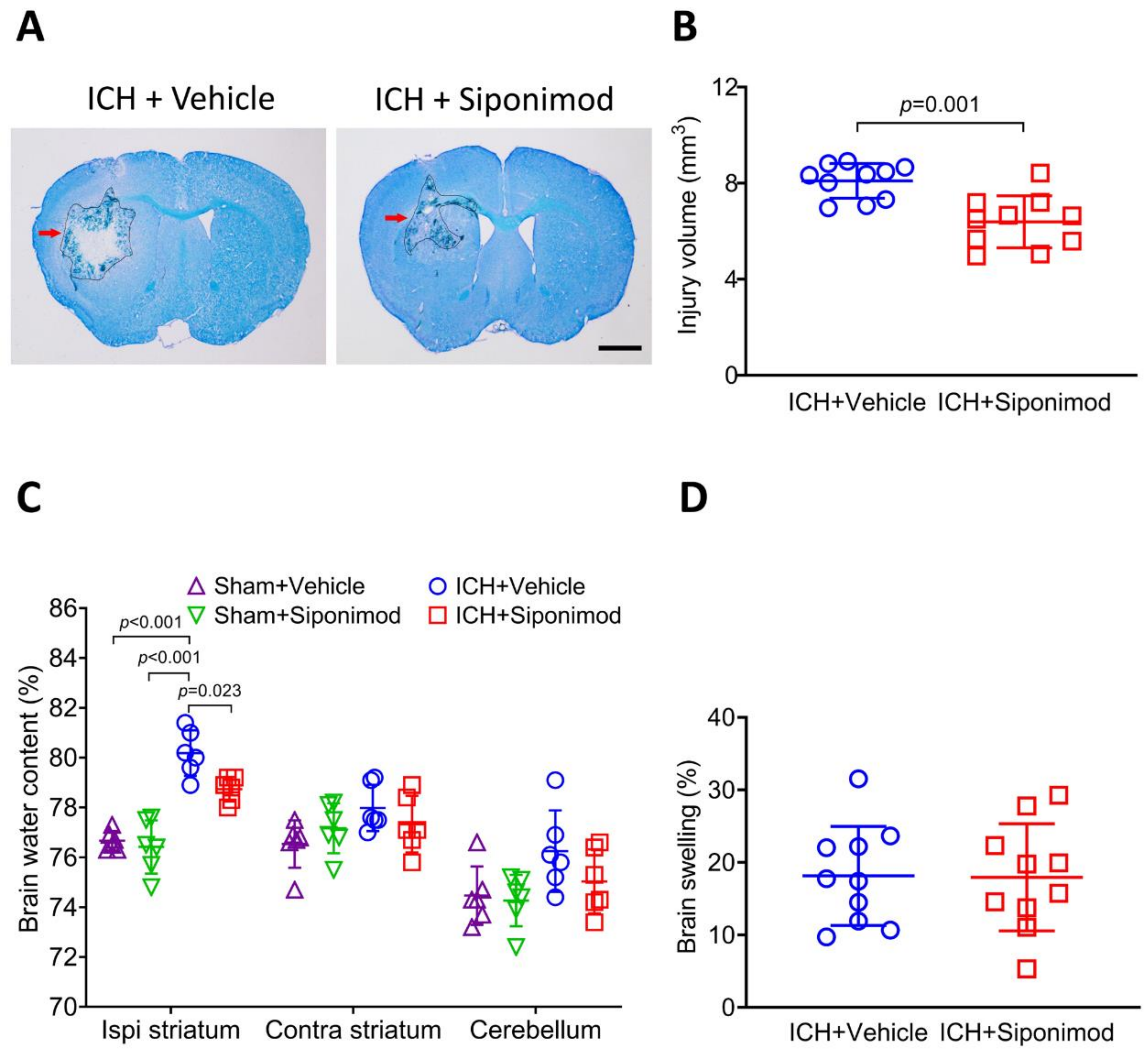
Siponimod treatment decreased brain injury volume and brain edema on day 3 after ICH. ICH was induced by injecting collagenase into the left striatum of C57BL/6 mice. (A) Representative brain sections were stained with LFB/CV on day 3. The areas of the lesion lacking staining are circled with a black curve (red arrow indicated); scale bar = 1 mm. (B) Brain injury volume was measured in LFB/CV-stained brain sections. The analysis revealed that siponimod treatment decreased brain injury volume compared to the vehicle-treated group on day 3 post-ICH. The *t*-test for the analysis of brain injury volume. *n* = 10 mice per group. (C) On day 3 after ICH, siponimod treatment decreased the water content of the ipsilateral striatum. One-way ANOVA followed by Bonferroni’s post hoc test for the analysis of brain water content. *n* = 6 mice per group. (D) Siponimod treatment did not influence brain swelling on day 3 after ICH. The *t*-test for the analysis of brain swelling. *n* = 10 mice per group. All data are expressed as mean ± SD. Ipsi: ipsilateral, Contra: contralateral.

**Figure 2.**
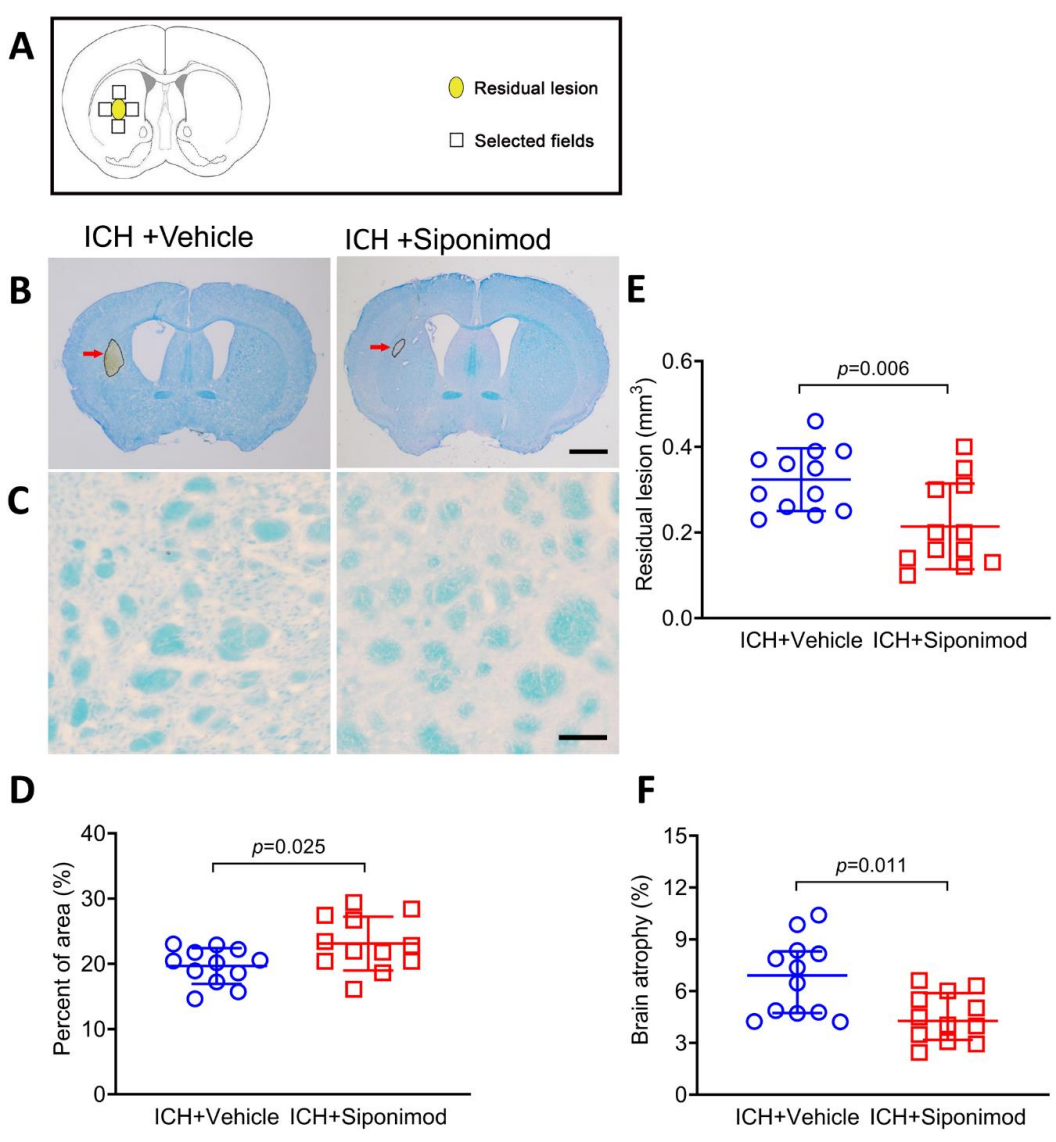
Siponimod treatment reduced the volume of brain lesions, myelin loss, and brain atrophy on day 28 after ICH. (A) The schematic diagram indicated where in the ICH brain for white matter damage measurements. (B) Representative images of brain sections stained with LFB/CV on day 28. The areas of the lesions are circled with the black curve (the red arrow indicated); scale bar = 1 mm. (C) LFB-stained myelin from brain sections in the perihematomal region on day 28. Scale bar = 1 mm. (D). Quantitative analysis of white matter damage. Treatment with siponimod reduced the loss of LFB-stained myelin in the perihematomal region. The *t*-test for the analysis of white matter damage. *n* = 12 mice per group. (E-F) Brain lesion volume and atrophy were measured in LFB/CV-stained brain sections. The scattergram shows the quantitative analysis of residual lesion volume (E) and brain atrophy (F). The *t*-test for the analysis of the volume of residual brain lesion, the Mann-Whitney U test for the analysis of brain atrophy. *n* = 12 mice per group. Data for brain atrophy are presented as median and IQR; other data are expressed as mean ± SD.

### Statistics

The sample sizes were determined with a power analysis (a power of 0.9 and a significance level of 0.05) [41]. Based on mortality and neurologic deficit score on day 28 after ICH as illustrated in one of our previous studies [41], power analysis showed that eight mice per group would be sufficient to detect a significant difference in corrected lesion volume on day 3. However, at least ten completed mice in each group could acquire a significant difference in neurologic deficit on day 28. To avoid the inadequacy of the sample size, we established a prespecified criterion to correct the sample size before this study was conducted [61, 76]. According to the requirements of the STAIR and the recommendations for the adjustment of expected attrition or death of animals, the following formula was used for the adjustment of the sample size: Corrected sample size = sample size / (1- [% attrition/100]) [61, 76]. In this study, expected attrition includes animals excluded (total NDS is less than 10 or more than 18 on day 1 after surgery) or those that died during the research period.

Statistical analysis was performed using GraphPad Prism 8.0.2. The distribution and homogeneity of the variance of each data set were evaluated using the Kolmogorov-Smirnov test and the homogeneity of the variance test. According to the distribution of each data set, all quantitative data were expressed as means ± standard deviation or median and IQR. Mortality was analyzed using a chi-square test. The difference between the two groups was diagnosed with a two-tailed Student's *t* test if the data were normally distributed and with the Mann-Whitney U test if not. One-way analysis of variance (ANOVA, parametric) or Kruskal-Wallis test (nonparametric) followed by Bonferroni’s post hoc test was used to check for changes between multiple groups. Repeated measures ANOVA was used to detect differences between treatment groups over time for body weight, rectal temperature, and corner turn test. Generalized estimation equations (GEE, nonparametric) were performed to evaluate NDS among multiple groups over time. Values of *p*<0.05 were considered statistically significant.

**Figure 3.**
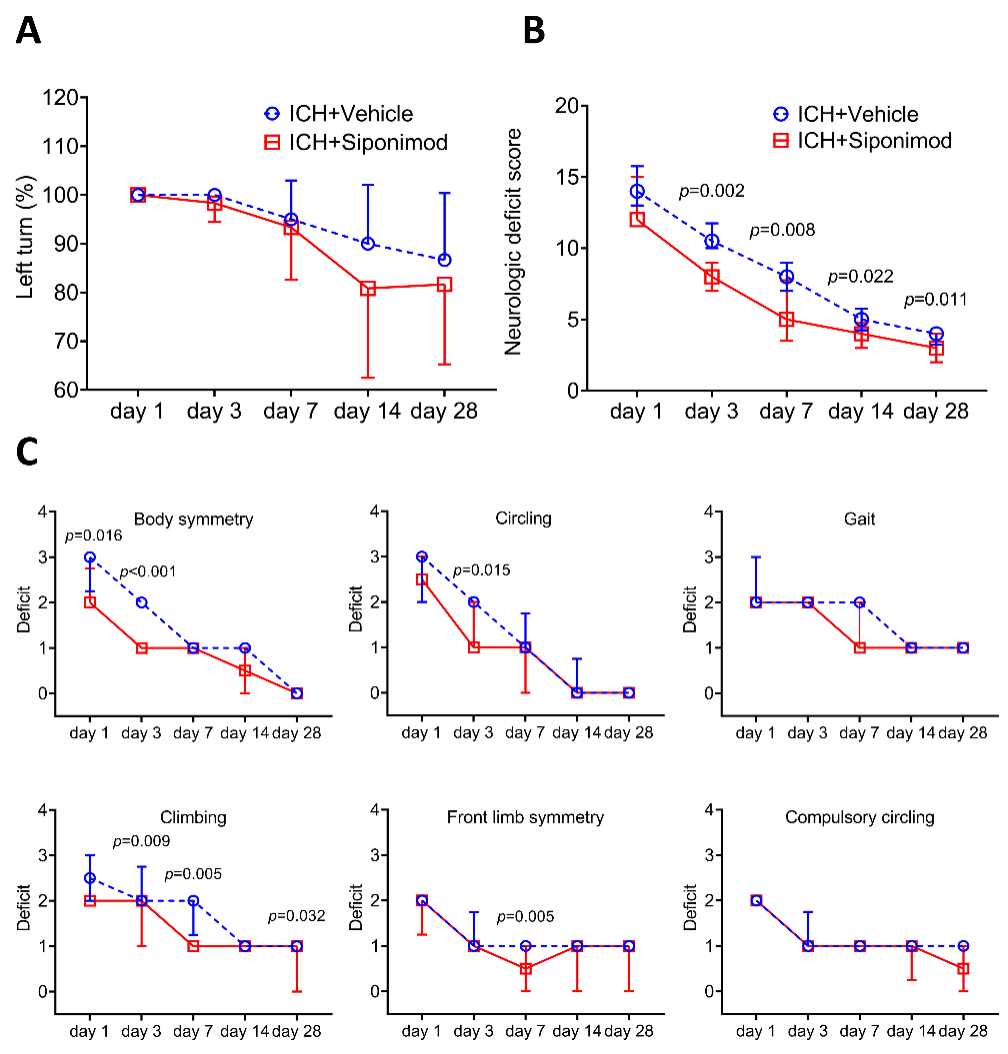
Treatment with siponimod improved long-term neurologic deficits after ICH. (A) The corner turn test (CTT) was not significantly different between the siponimod-treated and vehicle-treated groups (F = 0.659, *p* = 0.628; *p* > 0.05 at each time point). Repeated measures ANOVA followed by Bonferroni’s post hoc test for the analysis of CTT. *n* = 12 mice per group. (B) Compared to the vehicle-treated group, siponimod treatment improved neurologic function evaluated with neurological deficit scores (NDS) in mice on days 3, 7, 14, and 28 (Wald χ^2^ = 15.597, *p* < 0.001). Generalized estimation equations (GEEs) for the analysis of the NDS. *n* = 12 mice per group. (C) Neurological deficit scores for individual tests on days 1, 3, 7, 14, and 28 (generalized estimation equations, *n* = 12 mice per group). The CTT data are expressed as mean ± SD, and the NDS data are presented as median and IQR.

**Figure 4.**
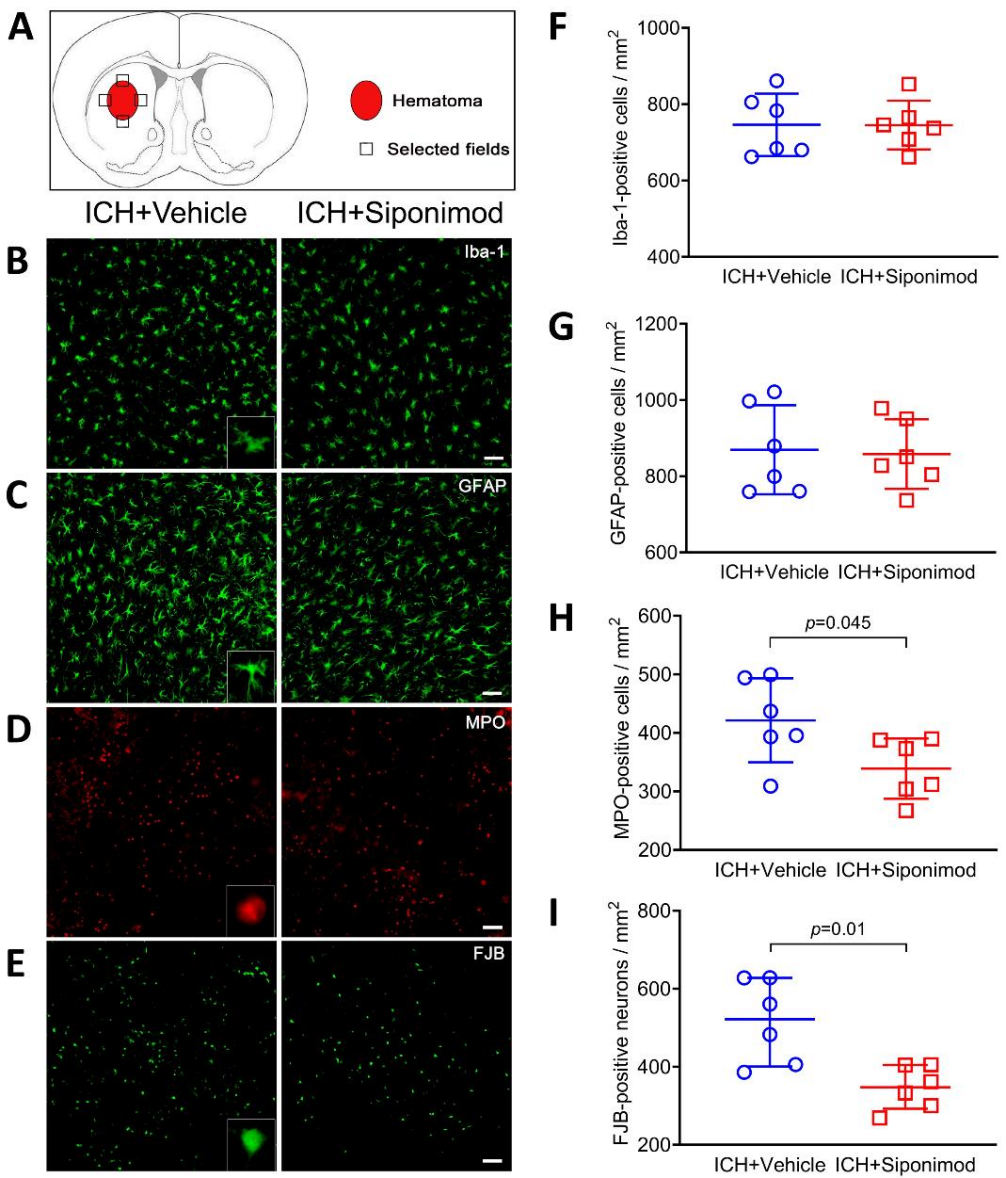
Siponimod treatment reduced neutrophil infiltration and the number of FJB-positive cells but did not influence microglia/macrophage or astrocytic activation on day 3 after ICH. (A) Schematic diagram of the selected fields for quantification of glial fibrillary acid protein (GFAP), ionized calcium-binding protein-1 (Iba-1), myeloperoxidase (MPO), and FJB-positive cells in 3 comparable sections of each mouse. (B-D) Representative images of immunofluorescence staining for Iba-1 (B), GFAP (C), and MPO (D) in the perihematomal region on day 3. The insets show a higher magnificence of immunofluorescence staining positive cells. Scale bar = 50 μm. (E) Representative images of histologic staining of FJB for degenerating cells on day 3. The inset shows the highest magnificence of FJB-positive cells. Scale bar = 50 μm. (F-I) Scattergrams show the quantitative analysis of Iba-1- (F), GFAP- (G), MPO- (H), and FJB- (I) positive cells. The *t-test* for the analysis of neutrophil infiltration and microglia/macrophage and astrocytic activation, and the Mann-Whitney U test for the analysis of FJB-positive cells. *n* = 6 mice per group. The data for FJB-positive cells are presented as median and IQR; other data are expressed as mean ± SD. Iba1: ionized calcium-binding adapter molecule 1, GFAP: glial fibrillary acid protein. MPO: myeloperoxidase, FJB: Fluoro-Jade B.

## RESULTS

### Siponimod does not affect mortality, body weight, or rectal temperature after ICH

Twenty-eight mice were excluded on day 1 after ICH. No sham mice died postoperatively in this study. In collagenase-induced ICH models, the mortality of siponimod-treated mice (10.52%, 4 out of 38) was similar to that of vehicle-treated mice (15%, 6 out of 40; *p* = 0.612) during the 28-day research period. Similarly, siponimod treatment did not affect the rectal temperature of mice from days 1 to 28 after ICH (F = 0.155, *p* = 0.958, *n* = 12 per group; Supplementary Fig. 1A). The decrease in body weight of ICH mice was observed in the first week after the onset of symptoms (Supplementary Fig. 1B). However, statistical analysis revealed that siponimod treatment did not change the body weight of mice compared to those that received vehicle intervention on days 1, 3, 7, 14, and 28 after ICH (F = 0.549, *p* = 0.702, *n* = 12 per group; Supplementary Fig. 1B).

### Siponimod decreased brain lesion volume and edema but did not affect brain swelling after ICH

LFB/CV staining was performed on day 3 after ICH to identify the effects of siponimod on brain lesion volume and swelling. Brain lesions were identified by areas lacking LFB/CV staining and destruction of myelin architecture (Fig. 1A). The analysis of the results showed that siponimod treatment significantly reduced the volume of brain lesions compared to vehicles treated ICH mice on day 3 after ICH (*t* = -4.133, *p* = 0.001, *n* = 10 per group; Fig. 1B). However, brain swelling, measured by the percentage of hemispheric enlargement, did not change after siponimod treatment compared to vehicle treatment after ICH (*t* = -0.06, *p* = 0.953, *n* = 10 per group; Fig.1D).

Brain edemas reflect impaired integrity of the blood-brain barrier (BBB), which can be used as a parameter to evaluate the severity of brain injury in ICH models. Therefore, we measured the brain water content of the ipsilateral, contralateral striatum, and cerebellum on day 3 after ICH. The results indicated that ICH caused a significant increase in water content in the ipsilateral striatum of mice in the vehicle-treated ICH group compared to those of the vehicle-treated sham-operated group (F = 32.442, *p*<0.001; sham surgery + vehicle *vs.* ICH + vehicle: *p* < 0.001; *n* = 6 per group, Fig. 1C). Further analysis also indicated that siponimod treatment significantly decreased brain water content in the ipsilateral striatum than in the vehicle-treated group on day 3 after ICH (ICH + siponimod *vs*. ICH + vehicle, *p* = 0.023; *n* = 6 per group, Fig. 1C). However, neither ICH nor siponimod influenced the water content in the contralateral striatum and cerebellum on day 3 after surgery (F values for the contralateral striatum and cerebellum were 2.096 and 2.827, respectively; *p* values for the contralateral striatum and cerebellum were 0.133 and 0.065, respectively, Fig. 1C).

### Siponimod treatment reduced residual lesion volume, myelin loss, and brain atrophy on day 28 after ICH

White matter injury is common in patients with ICH, usually resulting in poor outcomes and neurologic dysfunction after ICH [77]. To evaluate the influence of siponimod treatment on the severity of long-term brain injury, we measured the volume of residual lesions (Fig. 2B), brain atrophy (Fig. 2B), and myelin loss (Fig. 2C) with brain sections stained with LFB/CV or LFB from mice that received siponimod or vehicle treatment on day 28 after ICH. We found that, compared to the vehicle-treated group, siponimod significantly reduced myelin loss in the perihematomal region (*t* = 2.408, *p* = 0.025, *n* = 12 per group; Fig. 2D) and residual lesion volume and brain atrophy (*t* = -3.048, *p* = 0.006 for residual lesion; Z = -2.540, *p* = 0.011 for brain atrophy; *n* = 12 per group; Figs. 2E and F) on day 28 after ICH.

### Siponimod treatment improved long-term neurologic function of ICH

Long-term functional recovery can demonstrate stable protection compared to short-acting amelioration. For that reason, we evaluated neurologic function using modified NDS and CTT on days 1, 3, 7, 14, and 28 after ICH. NDS on day 1 after ICH did not differ between the vehicle treated group and the siponimod treated group, while mice treated with siponimod after ICH had lower NDS than vehicle treated ICH mice from days 3 to 28 after ICH (Wald χ^2^ = 15.597, *p* < 0.001; *p* = 0.207 on day 1, *p* = 0.002 on day 3, *p* = 0.008 on day 7, *p* = 0.022 on day 14, *p* = 0.011 on day 28; *n* = 12 per group; Fig. 3B). The results of individual tests are as follows: body symmetry on days 1 and 3, circling on day 3, climbing on days 3, 7, 28 and front limb symmetry on day 7 were significantly lower in the siponimod-treated group compared to those of the vehicle-treated group after ICH (*p* = 0.016 for body symmetry on day 1, *p* < 0.001 for body symmetry on day 3; *p* = 0.015 for circling on day 3; *p* = 0.009 for climbing on day 3, *p* = 0.005 for climbing on day 7, *p* = 0.032 for climbing on day 28; *p* = 0.005 for front limb symmetry on day 7; Fig. 3C). However, although there was a downward trend in the corner turn test score in siponimod-treated mice, no apparent differences between the siponimod and vehicle-treated group were found from days 1 to 28 after ICH (F = 0.659, *p* = 0.628, *n* = 12 per group; Fig. 3A).

### Siponimod treatment inhibited neutrophil infiltration and neuronal death, but did not activate microglia/macrophages or astrocytes in the peri-hematomal region

Immunofluorescence labeling of Iba1 and GFAP was used to examine the effects of siponimod on microglia/macrophage activation and astrocyte reactivity on day 3 after ICH. In this study, we define microglia/macrophage activation as cell bodies that were spherical, amoeboid, or rod-shaped in appearance, had a diameter of > 7.5 mm in at least one direction, and with short and thick processes exhibiting intense Iba1 immunoreactivity [41]. The resting microglia/macrophages were characterized by small cell bodies (< 7.5 mm in diameter) with long processes and weak Iba1 immunoreactivity [41]. Reactive astrocytes had more intense GFAP immunoreactivity with longer and thicker processes in the hemorrhagic hemispheres [41].

According to the morphological characteristics and cell body diameter of activated microglia/macrophages [41], we found that the number of activated microglia/macrophages in the perihematomal areas of mice was not significantly different between the siponimod- and vehicle-treated groups on day 3 after ICH (*t* = -0.021, *p* = 0.984, *n* = 6 per group; Fig. 4B and F). Similarly, based on the intense immunoreactivity of GFAP and the characteristics of the reactive astrocyte processes [41], we also found that siponimod treatment did not reduce the number of activated astrocytes on day 3 after ICH (*t* = -0.188, *p* = 0.855, *n* = 6 per group; Figs. 4C and G).

After ICH, neutrophils quickly arrive in the perihematomal areas and play a key role in the secondary brain injury process of ICH. We used MPO immunofluorescence labeling to examine the effect of siponimod treatment on neutrophil infiltration and found that fewer MPO-immunoreactive neutrophils were present in the hemorrhagic hemispheres of siponimod-treated mice than in vehicle-treated mice after ICH (*t* = -2.290, *p* = 0.045, *n* = 6 per group; Figs. 4D and H).

Next, we applied FJB staining to evaluate degenerated neurons around the hematoma on day 3 after ICH. We found that siponimod treatment also significantly reduced the number of FJB-positive cells in the perihematomal regions of ICH mice compared to vehicle-treated mice (Z = -2.562, *p* = 0.01, *n* = 6 per group; Figs. 4E and I).

**Figure 5.**
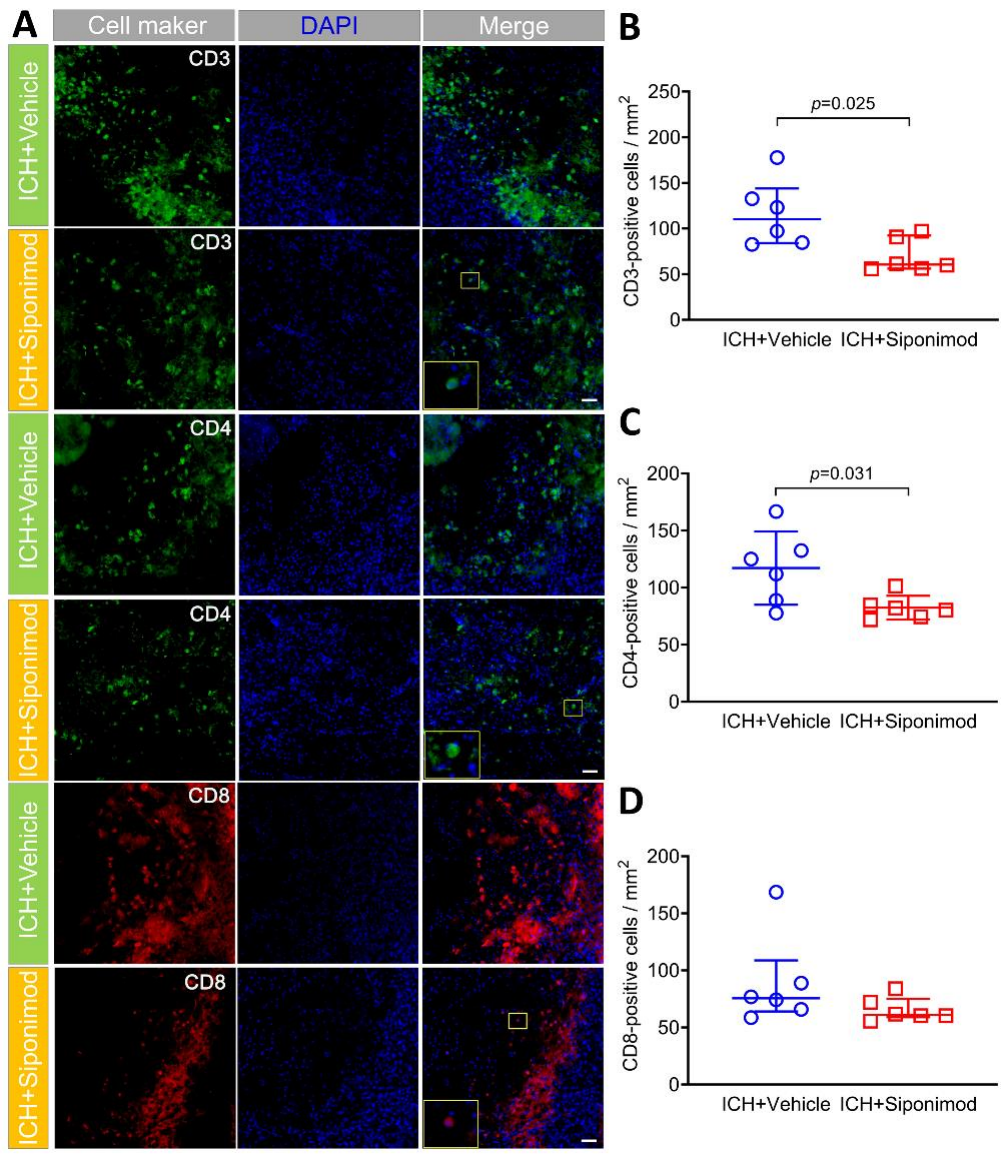
Siponimod treatment inhibited CD3^+^ and CD4^+^ lymphocyte infiltration in the perihematomal regions on day 3 after ICH. (A). The colonization of CD3, CD4, CD8, and DAPI in the perihematomal region on day 3 after collagenase-induced ICH. CD3 immunoreactivity is shown in green; CD4 immunoreactivity is shown in green; CD8 immunoreactivity is shown in red. Sections were stained with DAPI (blue) to label the nuclei. The insets show a higher magnificence of immunoreactive cells in the corresponding merged images. Scale bar = 50 μm. (B-D). Scattergrams show the quantitative analysis of CD3- (B), CD4- (C) and CD8- (D) positive cells. The selected fields in the 3 comparable sections of each mouse detected for lymphocyte infiltration are similar to Fig. 4. Mann-Whitney U test for the analysis of CD3^+^ and CD8^+^ lymphocytes, *t*-test for the analysis of CD4^+^ lymphocytes. *n* = 6 mice per group. Data for CD3^+^ and CD8^+^ lymphocytes are presented as median and IQR; other data are expressed as mean ± SD. DAPI: 4′, 6-diamidino-2-phenylindole.

### Siponimod treatment inhibited lymphocyte infiltration and reduced activated lymphocyte counts, but did not affect NK cell infiltration or activation of NK cells or CD3-negative immunocytes in the perihematomal regions

Lymphocyte infiltration promotes neuroinflammation and aggravates brain injury after acute ICH [14]. Here, CD3, CD4, and CD8 staining was used to identify T lymphocyte infiltration in perihematomal tissues. The results revealed that siponimod treatment reduced the number of CD3^+^ and CD4^+^ T lymphocytes in the perihematomal regions of ICH mice when compared to that in mice of the vehicle-treated group on day 3 after ICH (Z = -2.242, *p* = 0.025 for CD3; *t* = -2.514, *p* = 0.031 for CD4; *n* = 6 per group; Figs. 5A, B and C). Furthermore, siponimod treatment also reduced the number of CD8^+^ lymphocytes in the perihematomal regions of ICH mice when compared to that in mice of the vehicle-treated group on day 3 after ICH. However, the difference was insignificant (Z = -1.444, *p* = 0.149; *n* = 6 per group; Figs. 5A and D).

**Figure 6.**
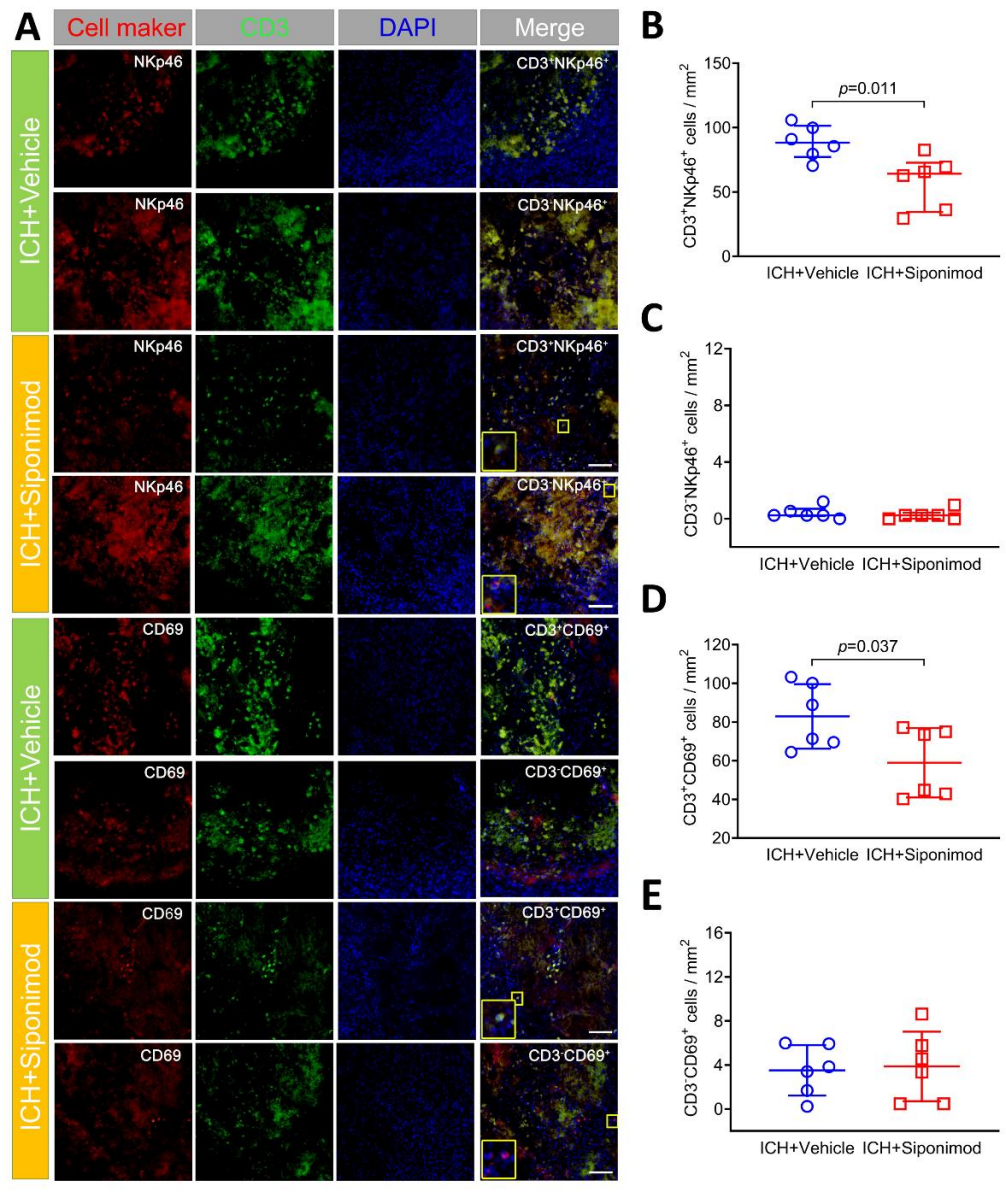
Siponimod treatment reduced the number of activated T lymphocytes in the perihematomal regions on day 3 after ICH (immunofluorescence). (A). Colocalization of CD3 and NKp46 and CD3 and CD69 in the perihematomal regions on day 3 after collagenase-induced ICH. CD3 immunoreactivity is shown in green; NKp46 is shown in red; CD69 immunoreactivity is shown in red. Sections were stained with DAPI (blue) to label the nuclei. The insets show a higher magnificence of immunoreactive cells in the corresponding merged images. Scale bar = 100 μm. (B-E). Scattergrams show the quantitative analysis of CD3^+^ NKp46^+^ (B), CD3^-^NKp46^+^ (C), CD3^+^ CD69^+^ (D) and CD3^-^CD69^+^ (E) cells. The selected fields in the 3 comparable sections of each mouse detected for lymphocyte activation are similar to Fig. 4. *t-test* for the analysis of CD3^+^NKp46^+^, CD3^+^CD69^+^, and CD3^-^CD69^+^ cells, Mann-Whitney U test for the analysis of CD3^-^NKp46^+^ cells. *n* = 6 mice per group. Data for CD3^-^NKp46^+^ cells are presented as median and IQR; other data are expressed as mean ± SD. DAPI: 4′, 6-diamidino-2-phenylindole.

**Figure 7.**
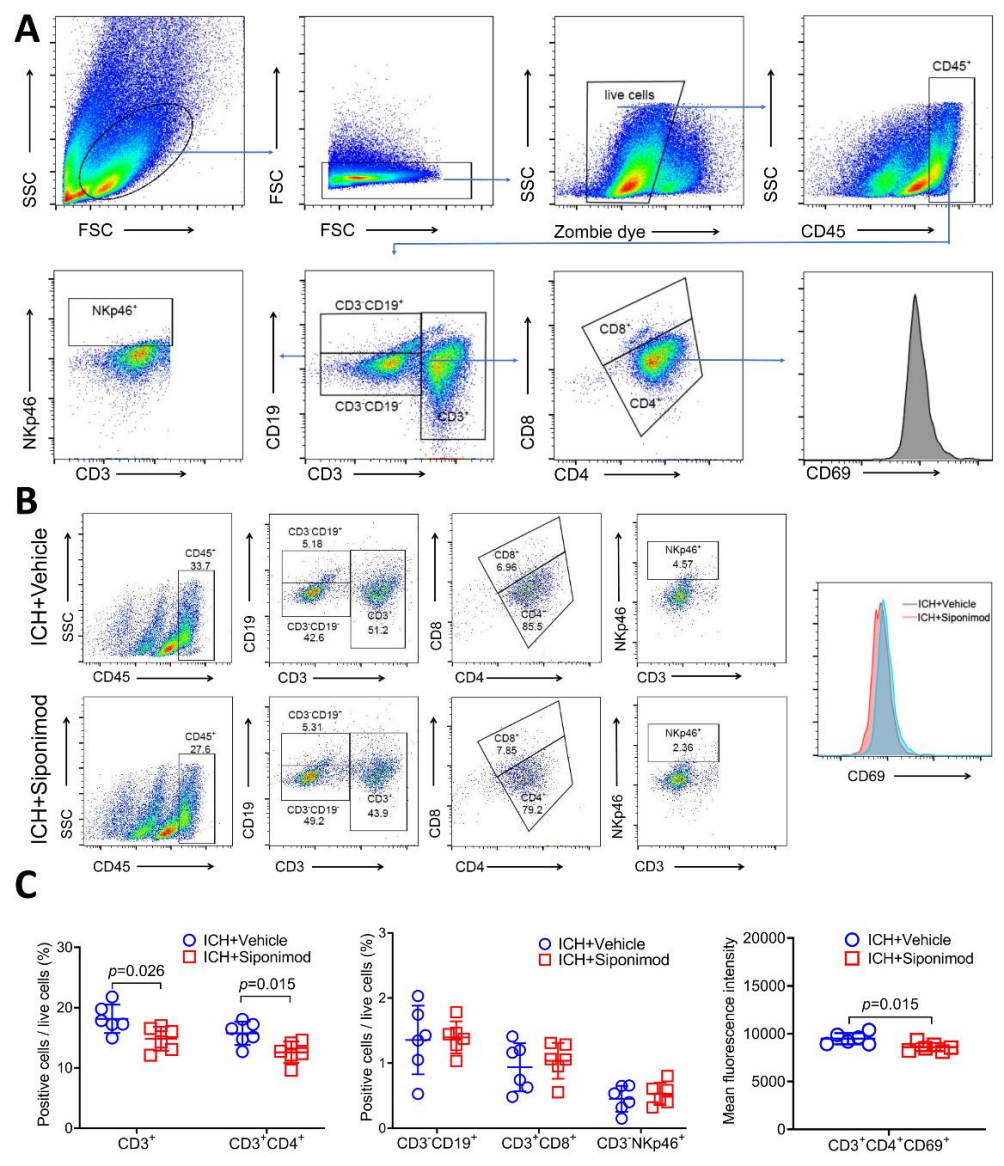
Siponimod treatment inhibited lymphocyte infiltration and alleviated Th cell infiltration and activation in the ICH brain on day 3 after ictus (Flow cytometry analysis). (A) Gating strategies for CD45^+^, CD3^+^, CD3^+^CD4^+^, CD3^+^CD8^+^, CD3^-^NKp46^+^, CD3^-^CD19^+^, and CD3^+^CD4^+^CD69^+^ cells. (B) Representative flow cytometry plot for CD45^+^, CD3^+^, CD3^+^CD4^+^, CD3^+^CD8^+^, CD3^-^NKp46^+^, CD3^-^CD19^+^, and CD3^+^CD4^+^CD69^+^ cells in brain samples from vehicle- and siponimod-treated mice on day 3 after ICH. (C) Percentages of lymphocytes, Th cells, CTLs, NK cells, and B lymphocytes and the MFI of CD69 in Th cells in vehicle- and siponimod-treated mice on day 3 after ICH (*t*-test, *n* = 6 mice per group). All data are expressed as mean ± SD.

To gain insight into the immunomodulatory effects of siponimod on ICH, we used double staining of CD3 and NKp46 or CD69 to evaluate the influence of siponimod on the infiltrated lymphocyte activation (CD3^+^NKp46^+^; CD3^+^CD69^+^) and infiltration and activation of NK cells (CD3^-^NKp46^+^; CD3^-^CD69^+^) [12, 78, 79]. Compared to the vehicle-treated group, siponimod reduced the number of activated lymphocytes (CD3^+^NKp46^+^; CD3^+^CD69^+^) in the perihematomal regions (*t* = -3.121, *p* = 0.011 for CD3^+^NKp46^+^; *t* = -2.397, *p* = 0.037 for CD3^+^CD69^+^; *n* = 6 per group; Figs. 6A, B, and D). Although a previous study found a substantial increase in infiltrating NK cells in the perihematomal regions of the animal and human brain 12 hours after ICH [12], our study found that only a few NK cells infiltrated the hemorrhagic brain of mice on day 3 after ICH. Furthermore, we also found that siponimod did not affect the number of infiltrated NK cells (CD3^-^NKp46^+^) (Z = -0.775, *p* = 0.438; *n* = 6 per group; Figs. 6A and C). In the design phase of this study, we were expected to detect the activation of NK cells with CD3^-^CD69^+^ in the hemorrhagic brain of mice. Interestingly, we found that CD3^-^CD69^+^ cells were more abundant in the perihematomal regions than CD3^-^NKp46^+^ cells, suggesting that CD3^-^CD69^+^ may not be the specific marker of activated NK cells. Other evidence has demonstrated that CD69 is also expressed by B lymphocytes, monocytes/macrophages, and neutrophils [80, 81]. Therefore, CD3^-^CD69^+^ only reflects the activation of CD3-negative immunocytes in this study. After analysis, we also found that siponimod did not reduce the counts of activated CD3-negative immunocytes (CD3^-^CD69^+^) in the hemorrhagic brain of mice on day 3 after ICH (*t* = 0.227, *p* = 0.825; *n* = 6 per group; Figs. 6A and E).

FACS analysis was also used for the detection of lymphocyte subpopulations in the hemorrhagic brain on day 3 after ICH. Representative flow cytometric dot plots in Fig. 7A show the detection strategies for lymphocytes, Th cells, CTLs, B lymphocytes, NK cells, etc. The percentages of infiltrated lymphocytes, Th cells, CTLs, B lymphocytes, NK cells, and the MFI of CD69 expression in Th cells in the ICH brain are shown in Figs. 7B and C. Similar to the immunofluorescence results, we found that siponimod treatment inhibited lymphocyte infiltration (CD45^+^CD3^+^) in the ICH brain on day 3 after ictus (*t* = 2.618, *p* = 0.026; *n* = 6 per group, Fig. 7 C). Furthermore, siponimod treatment also alleviated the percentages of infiltrated Th cells (*t* = 2.926, *p* = 0.015; *n* = 6 per group, Fig. 7C) in the hemorrhagic brain. However, it did not influence the percentages of infiltrated CTLs (*t* = -0.529, *p* = 0.609; n = 6 per group, Fig. 7C), B lymphocytes(*t* = -0.173, *p* = 0.866; *n* = 6 per group, Fig. 7 C) and NK cells (*t* = -0.710, *p* = 0.494; *n* = 6 per group, Fig. 7C). Regarding lymphocyte activation, we found that siponimod significantly reduced the mean fluorescence intensity (MFI) of CD69 expressed on Th cells (CD3^+^CD4^+^CD69^+^) in the hemorrhagic brain (*t* = 2.973, *p* = 0.015; *n* = 6 per group, Fig. 7C).

### Siponimod treatment decreased Th1-type cytokine production in the acute phase of ICH

**Figure 8.**
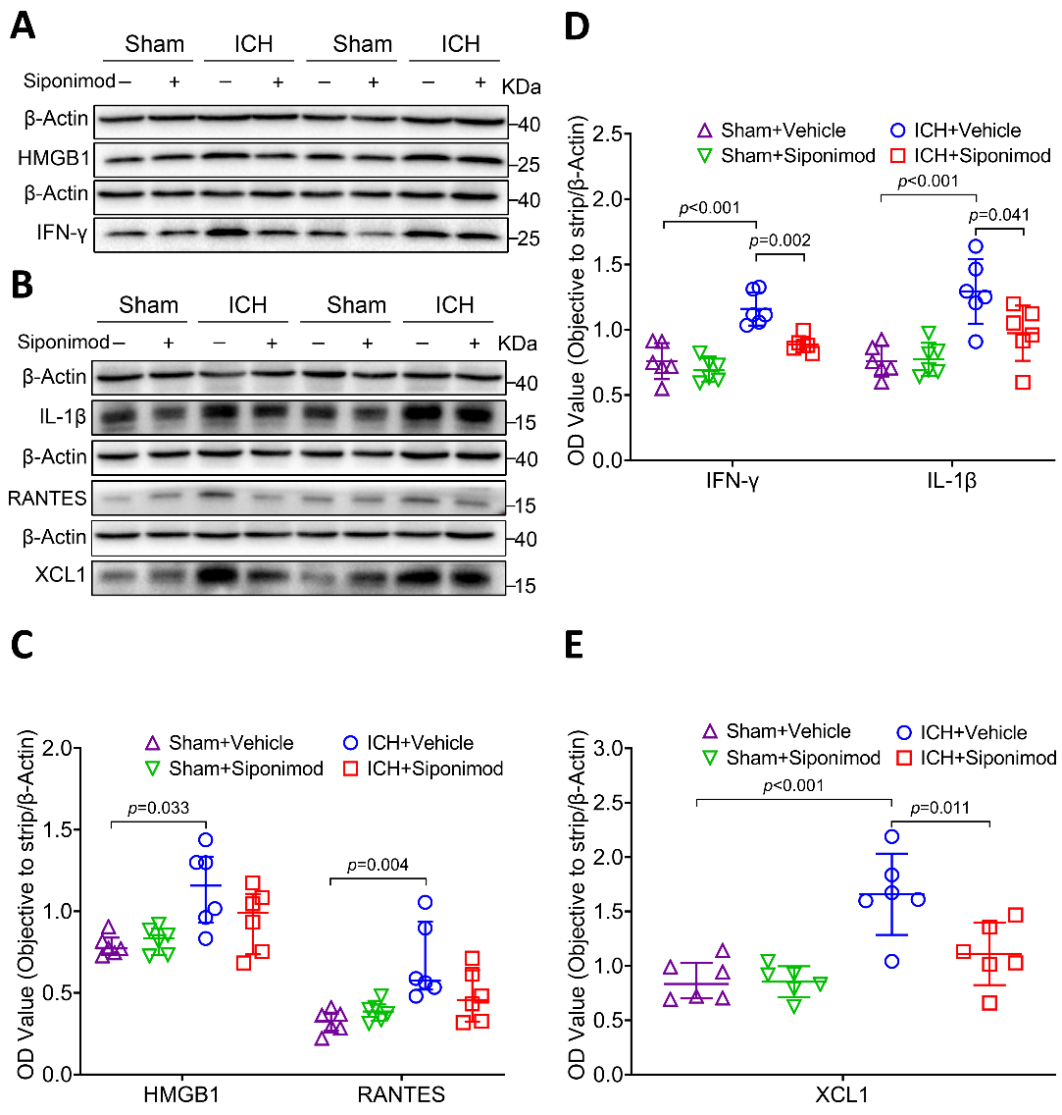
Treatment with siponimod decreased the expression of IFN-γ, IL-1β, and XCL1 at 36 h after ICH. (A-B) Representative Western blot bands of proinflammatory factor, HMGB1, IFN-γ, IL-1β, RANTES, and XCL1 were detected by loading brain protein samples from sham-operated and ICH mice treated with vehicle or siponimod, and β-actin was used as the loading control. (C-E) Scattergrams show the quantitative analysis of HMGB1, IFN-γ, IL-1β, RANTES, and XCL1 expression at 36 h after ICH. Densitometric quantification suggested that siponimod administration significantly decreased the levels of IFN-γ, IL-1β (D), and XCL1 (E) levels (one-way ANOVA followed by Bonferroni’s post hoc test, *n* = 6 mice per group), and siponimod treatment also reduced HMGB1 and RANTES (C) levels. However, it did not reach statistical significance (Kruskal-Wallis test for multiple comparisons, *n* = 6 mice per group). Data for HMGB1 and RANTES are presented as median and IQR; other data are expressed as mean ± SD. RANTES: On activation, normal T cells were expressed and secreted.

The cytokine HMGB1, Th1 inflammatory factors IFN-γ and IL-1β, and the chemokines RANTES and XCL1 may aggravate brain injury after acute stroke [11, 41, 82, 83]. The expression of HMGB1, IFN-γ, IL-1β, RANTES, and XCL1 was quantified by Western blotting 36 h after surgery (Figs. 8A and B; Supplementary Fig. 2). The results of the one-way ANOVA analysis and the Kruskal-Wallis test revealed that the expression of HMGB1, IFN-γ, IL-1β, RANTES, and XCL1 is different among the 4 groups (H = 9.493, *p* = 0.023 for HMGB1; F = 22.007, *p* < 0.001 for IFN-γ; F = 10.992, *p* < 0.001 for IL-1β; F = 12.620, *p* = 0.006 for RANTES; F = 12.264, *p* < 0.001 for XCL1; *n* = 6 per group; Figs. 8C-E). Further analysis with Bonferroni post hoc test of one-way ANOVA analysis and comparison among multiple Kruskal-Wallis test groups showed that the expression of HMGB1, IFN-γ, IL-1β, XCL1, and RANTES increased significantly at 36 h after ICH compared to sham operation in mice received vehicle treatment (*p* = 0.033 for HMGB1, *p* < 0.001 for IFN-γ, *p* < 0.001 for IL-1β, *p* < 0.001 for XCL1, *p* = 0.004 for RANTES, Figs. 8C-E). The results also indicated that siponimod significantly decreased the expression of IFN-γ, IL-1β, and XCL1 36 h after ICH compared to the vehicle treated group (*p* = 0.002 for IFN-γ, *p* = 0.041 for IL-1β, *p* = 0.011 for XCL1, Figs. 8D and E). Although there was a downward trend in HMGB1 or RANTES expression after siponimod treatment, the difference between the siponimod-treated group and the vehicle-treated group did not reach statistical difference at 36 h after ICH (*p* = 0.179 for HMGB1, *p* = 0.153 for RANTES, Fig. 8C).

### Anti-CD3 Abs alleviated the effects of siponimod on lymphocyte activation and Th1-type cytokine production in the acute phase of ICH

**Figure 9.**
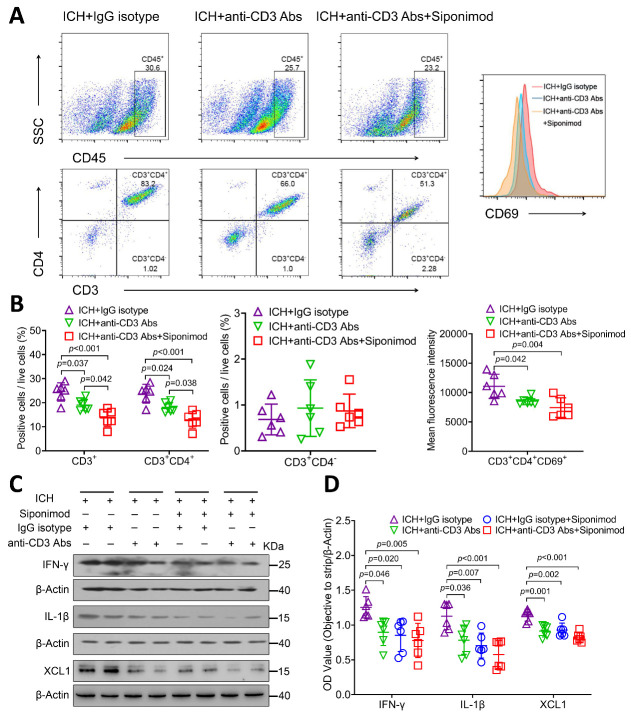
Anti-CD3 Abs alleviated the effects of siponimod on lymphocyte activation and Th1-type cytokine production in the acute phase of ICH. (A) Representative flow cytometry plots of CD3^+^, CD3^+^CD4^+^, CD3^+^CD4^-^, and CD3^+^CD4^+^CD69^+^ cells in the hemorrhagic brain of the ICH + IgG isotype group, the ICH + anti-CD3 Ab group, and the ICH + anti-CD3 Abs + Siponimod group on day 3 after ICH. (B) Percentages of lymphocytes, Th cells, CD3^+^CD4^-^ cells, and MFI of CD69 in Th cells in mice in the three groups on day 3 after ICH (one-way ANOVA followed by Bonferroni’s post hoc test, *n* = 6 per group). All data are expressed as mean ± SD. (C) Representative Western blot bands of IFN-γ, IL-1β, and XCL1 from mice of the ICH + IgG isotype group, the ICH + anti-CD3 Ab group, the ICH + IgG isotype control + Siponimod group, and the ICH + anti-CD3 Abs + Siponimod group. β-actin was used as a loading control. (D) Quantitative analysis of IFN-γ, IL-1β, and XCL1 expression 36 h after ICH (one-way ANOVA followed by Bonferroni’s post hoc test, *n* = 6 mice per group). All data are expressed as mean ± SD.

Induction of tolerance by anti-CD3 Abs has been tested for the control of the excessive inflammatory immune response, including the Th 1 immune response *in vivo* [84-87]. To further verify that siponimod exerts neuroprotective effects not only by inhibiting lymphocyte infiltration but also by downregulating lymphocyte activation after ICH, we investigated the effects of siponimod on lymphocyte activation and Th1-type cytokine production in an anti-CD3 Abs induced immune tolerance model with ICH. Mice in the ICH + IgG isotype control group, ICH + anti-CD3 Ab group, and ICH + anti-CD3 Abs + Siponimod group were included for the test. With FACS analysis, we found that the percentages of infiltrated lymphocytes (CD45^+^CD3^+^) (F = 15.858, *p* < 0.001; *n* = 6 per group, Figs. 9A and B) and Th cells (CD3^+^CD4^+^) (F = 17.340, *p* < 0.001; *n* = 6 per group, Figs. 9A and B) decreased significantly in hemorrhagic brains of the ICH + anti-CD3 Ab group (*p* = 0.037 for lymphocytes, *p* = 0.024 for Th cells; *n* = 6 per group, Fig 9B) and the ICH + anti-CD3 Abs + Siponimod group (*p* < 0.001 for lymphocytes, *p* < 0.001 for Th cells; *n* = 6 per group, Fig. 9B) than those in the ICH + IgG isotype control group on day 3 after ICH. Meanwhile, the percentages of infiltrated lymphocytes (CD45^+^CD3^+^) and Th cells (CD3^+^CD4^+^) were lower in the hemorrhagic brains of the ICH + anti-CD3 Abs + Siponimod group than those of the ICH + anti-CD3 Ab group on day 3 after ICH (*p* = 0.042 for lymphocytes, *p* = 0.038 for Th cells; Fig. 9 B). We also found that CD3^+^CD4^-^ cells only account for a smaller proportion of CD3-positive cells in the hemorrhagic brain on day 3 after ICH (Fig. 9A). Additionally, the comparison of infiltrated CD3^+^CD4^-^ cell counts between the three groups was not statistically different (F = 0.480, *p* = 0.628, *n* = 6 per group, Figs. 9A and B). Therefore, we did not further stratify or quantify CD3^+^CD8^+^ cells within CD3^+^CD4^-^ cells in this section. Regarding lymphocyte activation, the CD69 MFI of Th cells decreased markedly in hemorrhagic brains of the ICH + anti-CD3 Ab and the ICH + anti-CD3 Abs + Siponimod group than those of the ICH + IgG isotype control group on day 3 after ICH (F = 8.378, *p* = 0.004, *p*=0.042 for the ICH + anti-CD3 Ab group; *p* = 0.004 for the ICH + anti-CD3 Abs + Siponimod group; *n* = 6 per group, Figs. 9A and B), but the comparison between the ICH + anti-CD3 Ab group and the ICH + anti-CD3 Abs + Siponimod group (*p* = 0.728) was not statistically different. Combined with the findings on the influence of siponimod on lymphocyte activation, as previously reported, these findings suggest that siponimod may also exert neuroprotective effects by inhibiting Th cell activation.

With Western blotting, we also detected the influence of siponimod on molecular immune response in the presence or absence of anti-CD3 Abs-induced tolerance by assigning mice to the following four groups: ICH + IgG isotype control, ICH + anti-CD3 Abs, ICH + IgG isotype control + Siponimod, ICH + anti-CD3 Abs + Siponimod. The statistical analysis revealed that the expression of IFN-γ, IL-1β, and XCL1 in the ICH brain changed significantly among the four groups at 36 h after ICH (F = 6.154, *p* = 0.004 for IFN-γ; F = 8.849, *p* < 0.001 for IL-1β; F = 14.820, *p* < 0.001 for XCL1; *n* = 6 per group; Figs. 9C and D). Additional analysis revealed that the use of anti-CD3 Abs (*p* = 0.046 for IFN-γ, *p* = 0.036 for IL-1β, *p* = 0.001 for XCL1; *n* = 6 per group; Fig. 9D), IgG isotype control + siponimod (*p* = 0.020 for IFN-γ, *p* = 0.007 for IL-1β, *p* = 0.002 for XCL1; *n* = 6 per group; Fig. 9D), or anti-CD3 Abs + siponimod (*p* = 0.005 for IFN-γ, *p* < 0.001 for IL-1β, *p* < 0.001 for XCL1; *n* = 6 per group; Fig. 9D) all decreased the expression of IFN-γ, IL-1β, and XCL1 when compared to the use of the IgG isotype alone. However, no differences were found in mice between the ICH + anti-CD3 Ab group, the ICH + IgG isotype control + Siponimod group, and the ICH + anti-CD3 Abs + Siponimod group (IFN-γ: *p* = 1.000, IL-1β: *p* = 1.000, XCL1: *p* = 1.000 for the ICH + anti-CD3 Ab group *vs*. the ICH + IgG isotype control + Siponimod group; IFN-γ: *p* = 1.000, IL-1β: *p* = 0.476, XCL1: *p* = 0.543 for the ICH + anti-CD3 Ab group *vs*. the ICH + anti-CD3 Abs + Siponimod group; IFN-γ: *p* = 1.000, IL-1β: *p* = 1.000, XCL1: *p* = 0.313 for the ICH + IgG isotype control + Siponimod group *vs*. the ICH + anti-CD3 Abs + Siponimod group; *n* = 6 per group, Fig. 9C). These findings suggest that Siponimod inhibits Th1-type cytokine production in a lymphocyte-dependent manner in the acute phase of ICH.

## DISCUSSION

Previous studies have shown that S1PR modulators, including siponimod, exert neuroprotective effects by inhibiting lymphocyte recruitment and alleviating the inflammatory response after ischemic stroke or traumatic brain injury (TBI) [16, 36, 37, 42]. However, few studies address the immunomodulatory effect of siponimod on ICH. Therefore, this study aims to investigate the effect of siponimod on histopathologic and cellular inflammatory responses, Th1-type cytokine production, and long-term neurologic function in mice with ICH after a 3-day post-treatment regimen. Several novel findings are presented in this study: (1) siponimod treatment had no effect on mortality, rectal temperature, or bodyweight; (2) siponimod treatment reduced brain lesion volume, brain edema, and neuronal death on day 3; (3) siponimod treatment inhibited the production of XCL1 and Th1 inflammatory factors but had no influence on HMGB1 or RANTES production; (4) siponimod treatment reduced neutrophil infiltration but did not affect microglial or astrocyte activation on day 3; (5) siponimod treatment reduced the number of CD3^+^, CD4^+^ T lymphocytes and activated CD3^+^NKp46^+^ and CD3^+^CD69^+^ lymphocytes, but did not influence NK cell infiltration or activation of CD3-negative immunocytes (CD3^-^CD69^+^) in the perihematomal regions on day 3; (6) anti-CD3 Abs induced tolerance neutralized the role of siponimod on T lymphocyte response and Th1-type cytokine production in the acute phase of ICH; (7) post-ICH treatment with siponimod improved neurologic recovery from days 3 to 28; (8) post-ICH treatment with siponimod reduced residual lesion volume, brain atrophy, and myelin loss in the perihematomal region on day 28. This is the first report on the regulatory effects of siponimod on the infiltration or activation of different lymphocyte subpopulations and the Th1 molecular immune response after acute ICH.

After ICH occurs, neuroinflammation in the hemorrhagic brain will be amplified by the activation of microglia and astrocytes and the recruitment and infiltration of circulating immunocytes, including neutrophils, T lymphocytes, NK cells, etc. [3, 11, 47]. Specifically, S1P and S1PR have been found to be widely expressed by immunocytes [29-33]. Furthermore, S1P-S1PR signaling has been well characterized in immune trafficking and activation in innate and adaptive immune systems [27, 88]. S1PR-targeted strategies may represent a promising immunomodulatory method for stroke treatment [89]. Previous studies have illustrated that fingolimod (FTY-720, an analog of S1PR, the first generation of S1P modulators) alleviated brain injury or promoted functional recovery by reducing cerebral lymphocyte infiltration after ischemic or hemorrhagic stroke [17, 90-92]. However, as an unselective S1PR modulator, fingolimod has a long half-life [93]. Furthermore, it also has multiple adverse effects, including hypertension, macular edema, bradycardia, pulmonary toxicity, hepatotoxicity, and immunologic suppression [94, 95]. Therefore, further exploration of the effects of other S1P modulators on neuroinflammation is reasonable. Siponimod, a new selective modulator of S1PR_1_ and S1PR_5_ with a short half-life and a better safety profile, has gained considerable attention as an immunomodulatory agent for the treatment of ischemic or hemorrhagic stroke in animals [16, 36, 37]. However, the findings on the efficacy of siponimod in functional outcomes after stroke are currently inconsistent [16, 36, 37].

This study was designed in more detail to explore the therapeutic value of siponimod in ICH. Multiple administrations of siponimod at a dose of 0.3 mg/kg or 3 mg/kg body weight beginning 30 minutes after surgery, daily for 3 days, but not a single administration 30 minutes after surgery, significantly decreased circulating lymphocytes 72 h after ICH in mice [16]. However, a higher dose of siponimod may increase the occurrence of adverse reactions, such as changes in blood pressure, changes in heart rate, etc. [93, 96, 97]. Besides, as previously illustrated, the half-life period (t_1/2_) of siponimod is short (6-30 h in different species) which may allow rapid recovery in the frequency of circulating lymphocytes after the last dose of siponimod is administered [93, 96, 97]. As lymphocytes may also exert neuroprotective effects in the recovery phase of ICH [11], we selected to administer siponimod intraperitoneally at a dose of 1 mg/kg 30 min after surgery and subcutaneously 24 and 48 h after ICH in this study. Furthermore, additional evidence obtained from magnetic resonance imaging has indicated that siponimod administered intraperitoneally 30 min after the operation may not influence bleeding in the acute phase of ICH [16]. Therefore, we did not measure hemoglobin content in the hemorrhagic brain with a spectrophotometric assay in the vehicle and spionimod treated groups earlier after ICH. By evaluating the volume of brain injury with a combination of hematoma and secondary injury by histopathological staining on day 3 after ICH, our findings suggest that siponimod significantly alleviated the severity of short-term brain injury after ICH.

Although there has been evidence that indicated siponimod treatment attenuated the neurologic deficit on day 3 and improved the survival of mice on day 10 after ICH [16, 36], no studies have investigated the long-term neuroprotective effects of siponimod on ICH. In this study, we showed for the first time that siponimod treatment also reduced residual lesions, mitigated myelin loss, and inhibited brain atrophy on day 28 after ICH. Furthermore, although we found that siponimod failed to alleviate the deficit of the corner turn test and each NDS test, including body symmetry, gait, circling behavior, front limb symmetry, climbing, and compulsory circling at most time points evaluated in this study, it reduced total NDS over the 28-day research period. The above results suggest that the modest efficacy of siponimod in ICH warrants further validation.

Additional studies on the regulatory mechanisms of siponimod on the immunoinflammatory response may facilitate clinical translation for the ICH treatment. Evidence has suggested that S1PR down-regulation could reduce the egress of lymphocytes from the lymphoid organs to the lymphatic circulation and prevent the migration of lymphocytes from the periphery to the central nervous system [98, 99]. Additionally, S1PR modulators may be able to regulate the activation of microglia/macrophages and astrocytes through S1PR expressed on their surfaces [89]. Previous studies have indicated that S1PR modulators alleviated the severity of brain injury, probably by inhibiting lymphocyte recruitment after stroke [90, 92]. Our studies also illustrated that siponimod significantly inhibited the infiltration of T lymphocytes into the hemorrhagic brain of mice. However, with CD68 staining, a marker of activated and phagocytic microglia/macrophages, one study demonstrated that fingolimod did not alter the number of CD68-positive cells in the mouse brain of ICH, suggesting that it may not have a significant influence on microglial activation after ICH [100]. Based on the morphological changes of Iba-1-stained microglia/macrophages, we also did not find that siponimod could inhibit the activation of microglia/macrophages in the hemorrhagic brain of mice. Furthermore, we did not find that siponimod could significantly influence astrocyte activation in perihematomal tissues.

However, there have been other studies that showed that S1PR modulators suppressed the proinflammatory characteristics of microglia and astrocytes and exhibited neuroprotective effects by recognizing S1PR expressed *in vitro* and an animal model of experimental autoimmune encephalomyelitis [33, 101]. Additional evidence also implies that S1PR modulators limit the inflammatory response associated with microglia by polarizing microglia from the M1- to M2-like phenotype through the STAT3 pathway after bilateral carotid artery stenosis in mice [102]. A concentration-dependent effect of siponimod may interpret these inconsistencies in the regulation of microglia and astrocyte function [33, 103]. Therefore, the influence of siponimod on the role of microglia and astrocytes after ICH needs further investigation. Although CD68 may reflect the phagocytic characteristic of microglia/macrophages, it is also expressed by neutrophils and monocytes [104, 105]. Specifically, it may be better to further verify the effects of S1PR modulators on microglial activation by comparing their impact on the number of Iba-1 and CD68 double positive cells and Iba-1-positive cells with activated characteristics in morphology in the hemorrhagic brain.

Neutrophils also play a critical role in brain injury and ICH repair processes [65, 68, 106]. Inhibition of neutrophil infiltration into the hemorrhagic brain can alleviate neuroinflammation and attenuate brain edema and neurologic deficits after ICH [106]. Previous evidence has indicated that S1P could interact with S1PR in neutrophils to promote neutrophil recruitment and activation, improve immune response, and increase tissue damage [107, 108]. Like the effects of the selective S1PR_1_ modulator RP101075 on ICH [109], this study also found that siponimod reduced the number of neutrophils around the perihematomal region 72 h after ICH. There is also evidence that inhibition of the S1P pathway promoted the resolution of neutrophil inflammation *in vitro* [110]. According to the surface markers and cytokines released, neutrophils can be classified as phenotypes N0, N1, and N2. N1 neutrophils may exert neurotoxic effects by increasing the expression of proinflammatory cytokines, while N2 neutrophils may play neuroprotective effects by increasing lactoferrin secretion, which contributes to hematoma detoxification after ICH [111, 112]. Whether siponimod can enhance benefits and suppress adverse effects of neutrophils in the pathophysiological process of ICH needs further exploration.

T lymphocytes immediately infiltrated the brain parenchyma around the hematoma at 24 h and then peaked on day 5 following ICH and aggravated brain injury [14]. S1PR-dependent migration of T lymphocytes from secondary lymphoid organs into the lymphatic and blood circulation has been well studied [99, 113]. Studies on the immunomodulatory effects of S1PR after stroke focus mainly on modulation of S1PR on T lymphocyte migration from the periphery to the lesioned brain [90, 92]. With immunofluorescence staining, this study also found that siponimod significantly inhibited CD3^+^ and CD4^+^ T lymphocyte infiltration into the hemorrhagic brain. Furthermore, siponimod significantly reduced activated T lymphocytes (CD3^+^CD69^+^; CD3^+^NKp46^+^) in the perihematomal areas, implying that S1PR modulators may have multiple immunomodulatory effects.

Furthermore, our flow cytometric analysis further indicated that siponimod significantly reduced CD3^+^ CD4^+^ lymphocyte counts and the MFI of CD 69 in CD3^+^CD4^+^ lymphocytes in the perihematomal areas. Reduced counts of brain-infiltrated T lymphocytes may explain the reduction in activated T lymphocyte cells in the hemorrhagic brain after siponimod treatment. Additional studies should be conducted to explore whether siponimod can directly down-regulate the activation of infiltrated lymphocytes in the perihematomal regions.

Previous evidence showed that lymphocytes play a bidirectional role in the pathophysiological process of stroke, as T lymphocytes have different subpopulations [21]. For example, Treg cells can exert neuroprotective effects after ICH [114]. To illustrate the impact of S1PR modulation on the beneficial roles of lymphocytes, a study revealed that fingolimod increased Treg frequency in the spleen and blood after ischemia and increased the number of FoxP3^+^ cells in the ischemic brain of mice [115]. The novel immunomodulatory effect of S1PR on different subpopulations of lymphocytes warrants additional research. NK cells arrive in the hemorrhagic brain within 12 h after the onset of ICH, contributing to early PHE formation and exacerbating ICH injury [12]. To further understand the immunomodulatory effects of S1PR on stroke, our aim was to detect the effects of siponimod on the infiltration and activation of NK cells in the hemorrhagic brain, which were stained with CD3^-^NKp46^+^ and CD3^-^CD69^+^. However, our immunofluorescent staining results revealed that fewer NK cells (CD3^-^NKp46^+^) infiltrated the hemorrhagic brain on day 3 after ICH, contradicting the finding within 12 hours after ICH, as previously illustrated [12]. Although different time phases may explain the above difference, we demonstrated that the number of CD3^-^CD69^+^ cells is greater than the total NK cells in the hemorrhagic brain on day 3 after ICH. Combined with previous evidence on the expression of CD69 in B lymphocytes, monocytes/macrophages, and neutrophils [80, 81], we concluded that CD3^-^CD69^+^ could not precisely reflect NK cell activation. Therefore, we used CD3^-^CD69^+^ as a marker of activated CD3-negative immunocytes in this study.

Further analysis of immunofluorescent staining results revealed that siponimod did not significantly affect the infiltration of NK cells or the activation of CD3-negative immunocytes in the hemorrhagic brain on day 3 after ICH. With flow cytometric analysis, we also found that siponimod did not inhibit the infiltration of NK cells, CTLs, and B lymphocytes from blood to the hemorrhagic brain. The influence of siponimod on the infiltration and activation of different subpopulations also warrants further exploration after ICH.

CD4 is mainly expressed by Th cells [116]. The reduction in the frequency of CD4^+^ cell infiltration in this study suggests that siponimod may inhibit Th cell migration and infiltration from the periphery to the hemorrhagic brain. The Th1 immune response has been shown to have detrimental effects on the lesioned brain after ischemic stroke [117]. However, almost no studies have explored the efficacy of Th1 immune response on ICH. Thus, we evaluated the effectiveness of siponimod in the expression of Th1-type inflammatory factors, including IFN-γ and IL-1β in the hemorrhagic brain. The results indicated that siponimod significantly inhibited the expression of IFN-γ and IL-1β in the hemorrhagic brain. RANTES originates from various cells, including T lymphocytes, smooth muscle cells, endothelial cells, and glial cells, which could recruit and activate CD4^+^, CD8^+^, and NK cells [23, 82]. XCL1 is produced by subsets of T lymphocytes and NK cells during inflammation and is considered one of the triggers of secondary injury in traumatic brain injury [83]. RANTES and XCL1 can also drive the Th1 immune response [24, 25, 118]. However, we found that siponimod only inhibited the expression of XCL1 but did not affect RANTES in the hemorrhagic brain. The lack of effect on RANTES may be due to the diversity of its source cells, such as endothelial cells, glial cells, etc. However, this hypothesis needs to be further studied.

As an early proinflammatory mediator, HMGB1 can promote neuroinflammation and microglial activation and aggravate cerebral edema and brain injury after ICH [41]. This study also examined the regulatory effects of siponimod on the HMGB1-associated cellular inflammatory response. Interestingly, these results imply that they have no influence on HMGB1 expression in the hemorrhagic brain, suggesting that the siponimod-regulated Th1 immune response may be independent of HMGB1 post-acute ICH. More studies should be carried out to verify the regulatory effects of S1PR modulators on the cellular inflammatory response of the brain after ICH.

Evidence has indicated that anti-CD3 Abs can penetrate the BBB [119]. In this study, we designed an additional section to explore whether siponimod inhibited Th1-type cytokine production in a lymphocyte-dependent manner by inducing tolerance of T lymphocytes with anti-CD3 Abs [84-87]. Similar to some previous studies on the effects of anti-CD3 Abs on the cellular and molecular immune response in atherosclerosis, autoimmune diseases, etc. [84-87], our research provides evidence that anti-CD3 Abs downregulated lymphocyte infiltration and activation, and Th1-type cytokine production in the ICH brain. Although the combined use of anti-CD3 Abs and siponimod can further inhibit CD3^+^ and CD3^+^CD4^+^ lymphocyte infiltration than anti-CD3 Abs alone, they did not alleviate CD3^+^CD4^+^ lymphocyte activation (CD69 MFI) compared to anti-CD3 Abs after acute ICH. Furthermore, no apparent differences were found between mice treated with siponimod and anti-CD3 Abs in Th1-type cytokine production after ICH. These findings suggest that anti-CD3 Abs-induced tolerance abolished the effects of siponimod on Th cell activation and Th1-type cytokine production after acute ICH.

Based on evidence of the therapeutic value of siponimod for multiple sclerosis, the immunomodulatory effects of siponimod have been well observed [120]. Similar to two previous studies on the effects of siponimod on lymphocyte infiltration after acute ICH [16, 36], our results further supported the idea that siponimod can alleviate brain injury by inhibiting lymphocyte infiltration from the periphery to the hemorrhagic brain in the acute phase of ICH. Furthermore, we also illustrated that siponimod could alleviate CD3- and CD4-positive lymphocyte activation and Th1-type cytokine production in the hemorrhagic brain after acute ICH. Therefore, it is crucial to elucidate whether siponimod may be a potential candidate for stroke treatment by highlighting the molecular pathways involved in the siponimod-mediated immunomodulatory process, including downstream signals of S1PR and transcription factors of the Th1 immune response after ICH.

This study has several limitations. Although the immunomodulatory effect of siponimod has been linked to attenuating functional deficits after ICH, this study did not address the optimal dose or therapeutic time window of siponimod to treat ICH. To promote the probability of clinical translation, additional studies should be designed to investigate the therapeutic value of siponimod for ICH in a large time window (e.g, started 6 to 12 h after ICH). Age and gender differences can also affect ICH outcomes in patients [121, 122]. Validation in aged or female mice is needed to confirm the efficacy of siponimod for ICH. These are critical for designing immunomodulatory therapies with S1PR modulators for patients with ICH.

Furthermore, our findings did not reveal the modulatory effects of siponimod on microglia/macrophages and astrocytes after ICH. This result is different from other reports [101, 123]. Therefore, the influence of siponimod on microglia/macrophages and astrocytes should be interpreted with caution. It may be reasonable to further explore its immunomodulatory effects on glial cells *in vitro* and *in vivo.* The immune-inflammatory response may also exert neuroprotective effects in the pathophysiological process of ICH, especially in the recovery phase of ICH [11]. Although we observed the proinflammatory characteristics of infiltrated lymphocytes in the acute phase of ICH, we did not detect changes in the counts and function of infiltrated lymphocytes in the hemorrhagic brain at multiple time points. Further studies on the influence of immunomodulatory therapies on dynamic changes in the frequency and function of infiltrated lymphocytes may benefit understanding of the role of infiltrated lymphocytes in different phases of ICH.

Most studies have suggested that anti-CD3 Abs inhibit the immune-inflammatory response, probably by deleting T lymphocytes *in vivo* [84, 86, 124, 125]. Specifically, therapies targeting CD3 with anti-CD3 Abs may benefit the treatment of type 1 diabetes and autoimmune diseases [86, 126]. However, some studies indicated that anti-CD3 Abs could promote the immune-inflammatory response by stimulating CD3 in lymphocytes *in vivo* [127-129]. Although we believe that this study confirmed the inhibitory effects of siponimod on T lymphocyte activation and Th1-type cytokine production in the ICH brain based on a successful model of tolerance of T lymphocytes with anti-CD3 Abs, our results in this aspect still warrant further verification.

Stroke-induced immunosuppression can aggravate the prognosis of stroke by regulating the local immune-inflammatory response of the brain and increasing susceptibility to infection [11]. In addition, siponimod and anti-CD3 Abs (145-2C11) can also affect systemic immune responses by causing a reduction in peripheral lymphocytes and change in blood inflammatory biomarkers *in vivo* [84, 125, 127]. However, in this study, we did not investigate peripheral immune changes in mice that received siponimod and/or anti-CD3 Abs (145-2C11) after ICH. Furthermore, although the use of anti-CD3 Abs in this study was to further verify the effect of siponimod on the infiltration and function of T lymphocytes in the ICH brain, we did not evaluate its impact on brain injury or neurologic function. Additional studies on the effects of anti-CD3 Abs on brain injury and neurologic function after ICH may add new knowledge for immuno-modulatory therapy of ICH.

Together, we systematically investigated the immunomodulatory effects of siponimod on neuroinflammation and its therapeutic value in a mouse model of ICH. Our results illustrated that siponimod treatment could mitigate the severity of short- and long-term brain injury and improve long-term neurologic outcomes after ICH. Mechanistically, we found that siponimod inhibited neutrophil infiltration and T lymphocyte infiltration and reduced activated T lymphocyte counts in the hemorrhagic brain. Furthermore, siponimod may alleviate the Th1 immune response and Th1-type cytokine production in the hemorrhagic brain. However, it did not influence the infiltration and activation of NK cells or CD3-negative immunocytes in the hemorrhagic brain of mice. Combined with previous reports on the efficacy of S1PR modulators in the neuroinflammatory response and neurologic function after stroke [36, 90, 92, 100], siponimod, a selective modulator of S1PR_1/5_, may serve as a valuable therapeutic agent for the treatment of ICH. Further research on the causality between the immunomodulatory effects of siponimod and stroke outcome may help facilitate translational research in ICH.

## Supplementary Materials

The Supplementary data can be found online at: www.aginganddisease.org/EN/10.14336/AD.2022.1102.

## Data Availability

All relevant data from this study are included in the article and its supplementary files (raw Western blotting bands and FACS figures) or are available upon reasonable request from the corresponding author.
