# Supplementary material for: Lymphocyte-Related Immunomodulatory Therapy with Siponimod (BAF-312) Improves Outcomes in Mice with Acute Intracerebral Hemorrhage
Source: Aging Dis. 2023 Jun 1;14(3):966–91. doi: 10.14336/AD.2022.1102 (PMC10187706; doi:10.14336/AD.2022.1102)
Supplement: Supplementary file 1 — The Supplementary data can be found online at: www.aginganddisease.org/EN/10.14336/AD.2022.1102. [file AD-14-3-966-s.pdf]

SUPPLEMENTARY DATA

Lymphocyte-Related Immunomodulatory Therapy with Siponimod (BAF-312) Improves Outcomes in Mice with Acute Intracerebral Hemorrhage

Zhiying Zhang[1,2], Yinuo Li[1], Juyuan Shi[1], Li Zhu[1], Yinming Dai[1], Peiji Fu[1], Simon Liu[3], Michael Hong[4], Jiewen Zhang[5*], Jian Wang[1,6*], Chao Jiang[1*]

© 2022. Zhang Z et al. Published online at http://www.aginganddisease.org/EN/10.14336/AD.2022.1102

SUPPLEMENTARY DATA

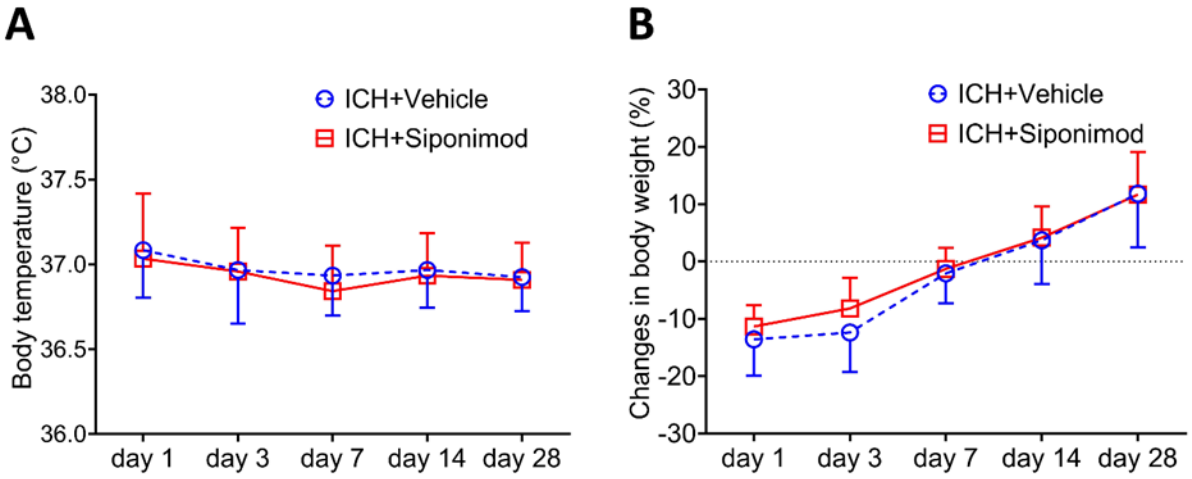

Supplementary Figure 1. Siponimod has no effect on rectal temperature or body weight after ICH. (A-B) Siponimod treatment did not affect rectal temperature (A) or body weight (B) compared to the vehicle-treated ICH group at any time during the 28-day research period. *n*=12 mice/group, all data are expressed as mean ± SD.

© 2022. Zhang Z et al. Published online at http://www.aginganddisease.org/EN/10.14336/AD.2022.1102

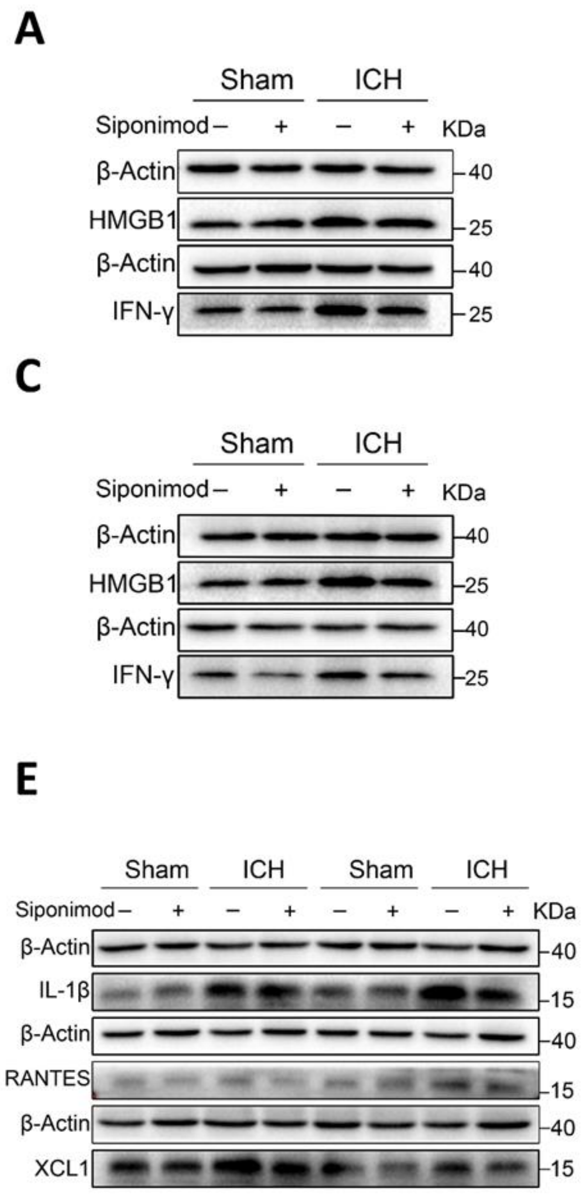

Supplementary Figure 2. Additional results for the expression of HMGB1, IFN-γ, IL-1β, RANTES, and XCL1 detected by Western blotting in Fig. 8 (A-F) Western blot bands of proinflammatory factors HMGB1, IFN-γ, IL-1β, RANTES, and XCL1 from 4 brain protein samples from ICH and sham-operated mice treated with vehicle or siponimod. The bands in Figure 8 combined with the bars in this figure were analyzed to evaluate the effect of siponimod on Th1-type cytokines in the hemorrhagic brain 36 h after ICH.

© 2022. Zhang Z et al. Published online at http://www.aginganddisease.org/EN/10.14336/AD.2022.1102

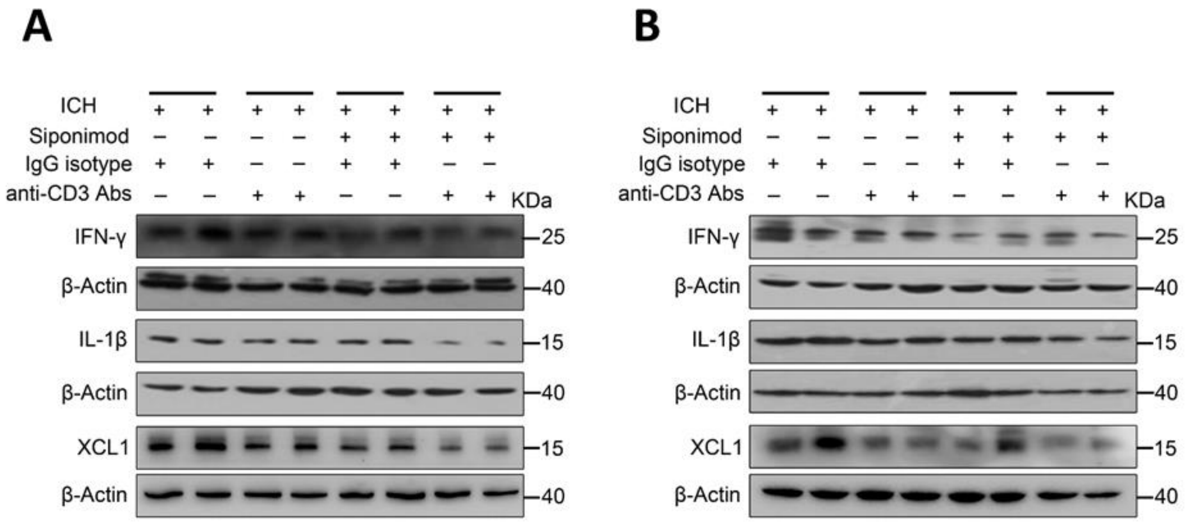

Supplementary Figure 3. Additional results for the expression of IFN-γ, IL-1β, and XCL1 detected by Western blot in Fig. 9 (A-B) Western blot bands of proinflammatory factors IFN-γ, IL-1β, and XCL1 from 4 brain protein samples of ICH mice treated with vehicle, siponimod, IgG isotype, or anti-CD3 Abs. The bands in Figure 9 combined with the bars in this figure were analyzed to assess whether anti-CD3 Abs can alleviate the effects of siponimod on Th1-type cytokines in the hemorrhagic brain 36 h after ICH.

© 2022. Zhang Z et al. Published online at http://www.aginganddisease.org/EN/10.14336/AD.2022.1102
